# Conscious Perception as Integrated Information Patterns in Human Electrocorticography

**DOI:** 10.1523/ENEURO.0085-17.2017

**Published:** 2017-10-04

**Authors:** Andrew M. Haun, Masafumi Oizumi, Christopher K. Kovach, Hiroto Kawasaki, Hiroyuki Oya, Matthew A. Howard, Ralph Adolphs, Naotsugu Tsuchiya

**Affiliations:** 1 Department of Psychiatry, University of Wisconsin-Madison, Madison, WI; 2 School of Psychological Sciences, Monash University, Clayton, Australia; 3 RIKEN Brain Science Institute, Wako, Japan; 4 Department of Neurosurgery, University of Iowa, IA; 5 Division of Humanities and Social Sciences, California Institute of Technology, Pasadena, CA; 6 Monash Institute of Cognitive and Clinical Neuroscience, Monash University, Clayton, Australia

**Keywords:** consciousness, electrocorticography, face perception, integrated information theory

## Abstract

A significant problem in neuroscience concerns the distinction between neural processing that is correlated with conscious percepts from processing that is not. Here, we tested if a hierarchical structure of causal interactions between neuronal populations correlates with conscious perception. We derived the hierarchical causal structure as a pattern of integrated information, inspired by the integrated information theory (IIT) of consciousness. We computed integrated information patterns from intracranial electrocorticography (ECoG) from six human neurosurgical patients with electrodes implanted over lateral and ventral cortices. During recording, subjects viewed continuous flash suppression (CFS) and backward masking (BM) stimuli intended to dissociate conscious percept from stimulus, and unmasked suprathreshold stimuli. Object-sensitive areas revealed correspondence between conscious percepts and integrated information patterns. We quantified this correspondence using unsupervised classification methods that revealed clustering of visual experiences with integrated information, but not with broader information measures including mutual information and entropy. Our findings point to a significant role of locally integrated information for understanding the neural substrate of conscious object perception.

## Significance Statement

What is the link between neural activity and conscious experience? It is clear that experience is generated in the brain, as conscious experience occurs even without sensory inputs, but it is also clear that not everything that occurs in the brain is correlated with consciousness. There must be some phenomenon occurring in brains that is critical for consciousness. In this article, we tackle this issue from a new direction: starting from conscious phenomenology, we derive a novel measure of distributed population neural activity, the integrated information pattern, and find that, when applied to intracranial field potential recordings (electrocorticography, ECoG), this measure can be used to classify the conscious perceptual experiences of human subjects.

## Introduction

**Figure 1.**
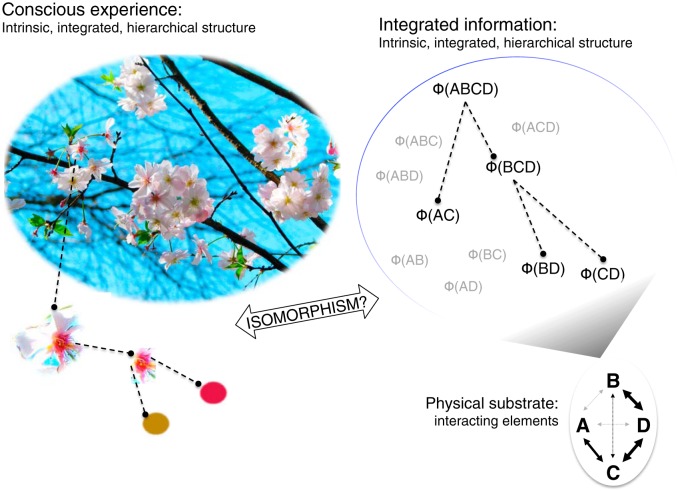
Left, Conscious experience is a multilevel integrated pattern, where, for example, distinct colors constitute shapes, which constitute distinct objects, which constitute distinct scenes, which are part of a multimodal conscious percept. Right, A system of interacting elements (ABCD, lower right corner) generates a multilevel pattern of integrated information. In this example, an integrated system ABCD is supported by AC and BCD, with the latter further supported by BD and CD. IIT proposes that such a pattern of integrated information is isomorphic to the structure of a conscious experience.

The contents of conscious experience include our momentary perceptual, emotional, and cognitive experiences, what we are experiencing at this moment. These contents are bound together in a nested, compositional structure (Fig. 1). For example, certain colors and contours are parts of certain surfaces, which are parts of certain objects, which are parts of a certain visual scene, which is part of a particular multimodal moment of conscious experience as a whole. Any particular experience is also highly informative in the sense that it takes on a very specific form that excludes an enormous number of other possible experiences. These seem to be fundamental properties of any conscious experience: a highly informative set of many nested parts that are bound into a unified whole.

What could explain how the brain supports the compositional, bound and informative nature of the perceptual aspects of conscious experience? Integrated information theory (IIT; Tononi, 2004, 2012) provides just such a set of explanatory concepts. First, IIT considers the capacity of the brain for state differentiation, which corresponds to the informative nature of conscious experience. The larger the number of distinct states the brain can have, the more informative any particular state must be. Second, IIT quantifies integration of this information across a system by considering how much information is lost if system is partitioned by disconnecting its parts from one another. Third, IIT claims that different parts of an integrated system will play distinct roles in specifying its total information, and that these roles correspond to parts of conscious experience as a whole. More specifically, IIT proposes that it is the structure of a system’s integrated information (Fig. 1), the way its parts contribute to its total integration, that corresponds to the exact nature of the brain’s conscious contents.

**Figure 2.**
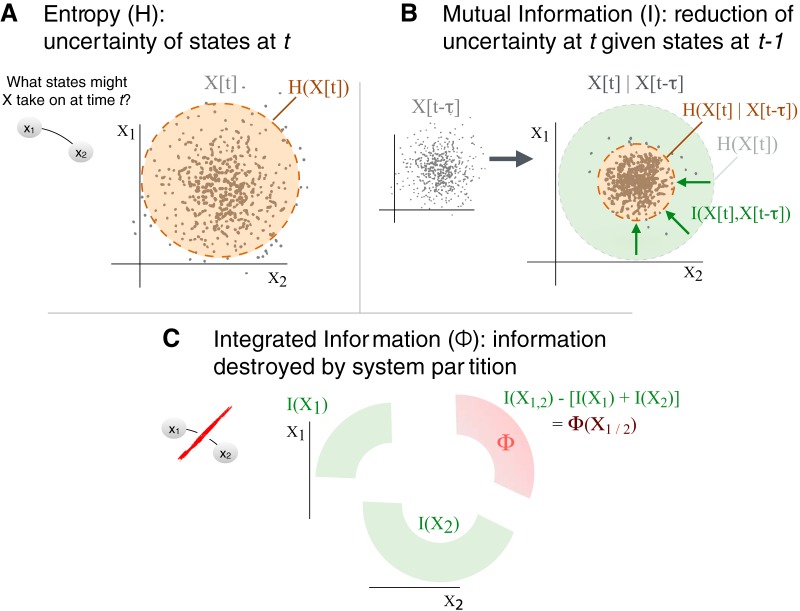
Derivation of integrated information for a “simplest” system of two data channels, X_1_ and X_2_, depicted by the two connected nodes on the far left. We assume that X_1_ and X_2_ can take continuous values at each time. ***A***, Entropy is the uncertainty of the system's states at time *t*, corresponding to its spread. X[t] is represented as a cloud of dots, a random distribution of joint values of X_1_ (state of unit 1) and X_2_ (state of unit 2). ***B***, Mutual Information. If states at time *t-τ* (X[*t-τ*]) are given, then uncertainty about the state at time *t* is reduced; this reduction, illustrated by the green “shrinkage” of the entropy, is called the Mutual information (I). ***C***, Integrated Information. Mutual information can be measured for the whole system, *I(X_1, 2_)*, and for each part of a “cut” system, *I(X_1_)* and *I(X_2_)*. Note we dropped *t* and *τ* for simplification. If the information of the whole system is greater than the sum of the information in the parts of the cut system, the residual is Integrated Information (Φ*), information generated only by the whole system. Note that this formulation is only for an intuitive illustrative purpose. We estimate the sum of the information of the cut systems using a more sophisticated method of “mismatched decoding,” which guarantees that 0 <= Φ* <= I (Oizumi et al., 2016a).

One limitation of the IIT account is that it has been developed in the context of simple model systems, and is computationally intractable for empirically observed neural data (Balduzzi and Tononi, 2008; Barrett and Seth, 2011). An IIT-driven analysis of neural activity thus requires approximated measures of integrated information. One such approximation is derived via the mutual information between time-lagged system states (Barrett and Seth, 2011; Oizumi et al., 2016a). Figure 2 illustrates this derivation. The entropy *H*(*X*(*t*)) of a multivariate system over a time interval quantifies state uncertainty (the state distribution breadth; Fig. 2*A*). Mutual information (or predictive information (Bialek et al., 2001) is computed across a short time lag as *I*(*X*(*t*);*X*(*t-τ*)): how is the state uncertainty reduced given states *X*(*t-τ*) in the past (Fig. 2*B*)? However, mutual information alone does not reflect integration across the system. To evaluate integration we partition the system, estimate *I* only within the parts, and recombine these estimates. The integrated information Φ is the divergence of the “true” *I* and the “partitioned” *I* (Fig. 2*C*), where the partition is chosen to minimize the divergence, making Φ a measure of the irreducibility of the mutual information. Importantly, Φ is uniquely defined for all parts of a system. For example, the integrated information in a system of three elements, A, B, and C, is exhaustively characterized as the set {Φ(AB), Φ(AC), Φ(BC), Φ(ABC)}; each member of this set identifies some aspect of the system’s integration. We refer to this set of Φ values as the integrated information pattern or Φ-pattern.

IIT proposes that the structure of a conscious perceptual experience is isomorphic to the integrated information generated in the brain (Fig. 1). If this is true, any change in conscious perception should exactly correspond to change in the pattern of integrated information: if two perceptual experiences are phenomenally similar, they should also be close in the similarity space of integrated information patterns. We tested this hypothesis by measuring Φ-patterns in human intracranial electrocorticography (ECoG) during various distinct visual experiences. The primary manipulation we used in this study is continuous flash suppression (CFS; Tsuchiya and Koch, 2005), which allows dissociation of physical stimulus and conscious perceptual contents. We buttressed our conclusions by extending our analysis to related but distinct paradigms of backward masking (BM) and unmasked stimulus presentation tested in the same subjects. For each stimulus/percept condition we extracted the Φ-pattern from groups of ECoG channels, and gauged the similarity between the patterns and the conscious contents. For comparison, we also extracted mutual information and entropy patterns, which are also compositional information patterns (Fig. 2) but do not reflect integration. We found that the Φ-patterns mapped onto percepts better than mutual information or entropy patterns. In some patients for whom there were electrodes in object-sensitive areas within the fusiform gyrus, the mapping could be extremely precise and consistent even across multiple stimulus paradigms. We propose that our results support the hypothesized isomorphism between the structure of conscious experience and the integrated information pattern generated by the human brain.

## Materials and Methods

### Subjects

We analyzed ECoG recordings obtained in six patients undergoing video EEG monitoring with intracranial electrodes. All patients had “grid” arrays installed over the left (*N* = 4) or right (*N* = 2) lateral temporal lobes, and five also had two or more “strip” arrays installed ventrally on the same side. Patients also had frontal and deep electrodes, which we did not include in our analyses. Recording was not performed within 48 h of major seizures. For the statistical analysis reported in Results, we regarded each row in Table 1 (each combination of subject, experiment paradigm, and lateral/ventral electrode array) as an independent condition. The Institutional Review Board at University of Iowa approved the study (approval number 200112047), and written informed consent was obtained from each patient.

**Table 1.**
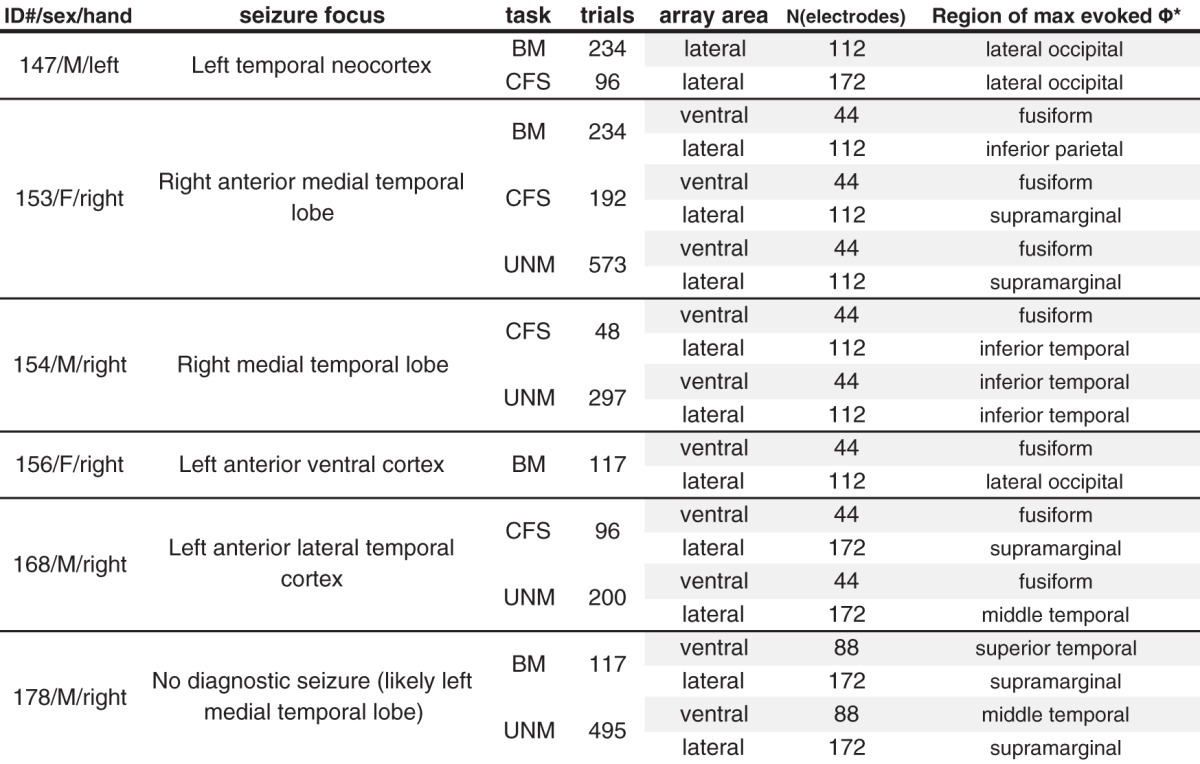
Patient demographics, electrode settings, experiment conditions, and evoked Φ* regions

### Psychophysics

Subjects performed psychophysics experiments during ECoG recording. All subjects were naïve to the tasks, and generally received a block or two of practice trials before data collection began. Completed trial counts for all included subjects are listed in Table 1. More detailed analyses of the tasks (CFS and unmasked tasks) are described elsewhere (Baroni et al., 2017).

**Figure 3.**
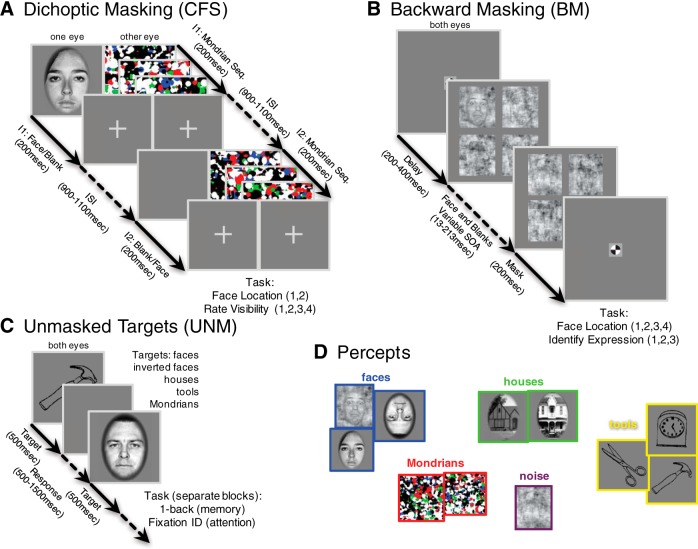
Paradigms for stimulus presentation. ***A***, CFS task. Each trial consisted of a temporal sequence of two stimulus intervals, separated by a random interstimulus interval (ISI, 900–1100 ms). In one eye, each interval contained three flashes of colorful Mondrian patterns; in the other eye, one interval contained a face image of variable contrast. These conditions result in stochastic trial-to-trial visibility of the target face; sometimes the face is consciously seen, sometimes it is not. The subjects’ task was to select which interval contained the face, and to indicate how visible it was on a scale of 1–4. ***B***, BM task. After a random fixation delay, subjects saw an array of four noise patches, one of which contained a face image (the upper left, in the illustration), for 13 ms. After a variable SOA, another array of noise patches was presented to reduce the visibility of the face. The subject’s task was to identify the location of the face target among four possible locations, and also to identify its emotional expression among three possible labels (happy, fearful, and neutral). ***C***, Unmasked conditions included a one-back memory task, in which subjects paid attention to the category of the stimuli, or a simple fixation task, in which they detected a change of color orientation of the fixation cross. In both tasks, faces and other objects were presented for 500 ms without any masks, with trials separated by a blank interval (500 ms for the fixation task, 1000 ms for the one-back task). ***D***, From subjects’ performance on a task (correct/incorrect, ratings of visibility, identification of expression), we can reasonably infer their percept on each trial of an experiment. We used subjects' performance to divide trials into the percept categories shown here: faces (and inverted faces), houses, tools, Mondrians, and noise.

The primary task was CFS, where a target face stimulus is presented to one eye while colorful Mondrian (shape noise) images are continuously flashed to the corresponding position in the other eye (Tsuchiya and Koch, 2005). Subjects fixated the stimuli through a custom-made 4-mirror stereoscope. Stimuli were presented on a 19-inch ViewSonic VX922 LCD display (75 Hz refresh rate). Face/Mondrian images subtended ∼7.5 × 10° in visual angle. We controlled the experiment and stimuli using Psychtoolbox (Brainard, 1997; Pelli, 1997) with MATLAB (version 7.8) on a PC running Windows XP. In each trial, two temporal intervals were presented (Fig. 3*A*). Each interval lasted 200 ms, and the two intervals were separated by a random duration between 900 and 1100 ms. In one interval, the target face was presented to one eye; in the other interval, the blank gray field was presented. In both intervals, three distinct patterns of Mondrians were presented (each 66 ms, at 15 Hz) to the other eye.

**Figure 4.**
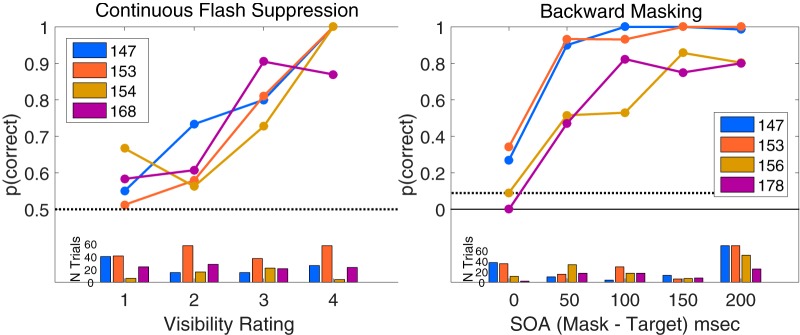
Behavioral performance on the CFS and BM tasks. Subjects who participated in each task are identified in the legend. Upper panels show proportion correct for each task. Lower panels show the number of trials at each trial type. Left, Proportion correct for four CFS subjects, as a function of visibility rating. Trials rated as 3 or 4 were treated as visible in the classification analyses. Right, Proportion correct for BM subjects, as a function of (binned) backward-mask SOA. Trials where subjects were correct on both 4AFC location and 3AFC emotion judgments (here coded as “correct,” making chance level 1/12) were treated as visible in the classification analyses. Catch trials were not included in the graph.

After the two intervals, the subject was asked to report which interval contained the target face, and then to report the subjective visibility of the target (a four-point scale ranging from “clearly visible” to “invisible”; Ramsøy and Overgaard, 2004), using keys on a USB keypad. Three target contrasts (either 50%, 25%, and 12.5% or 100%, 50%, and 25%, depending on the subject) were interleaved from trial to trial. The flashing Mondrian tended to suppress the target face, especially when the target contrast was lower. As a screening step (to ensure that included subjects understand the task and responded properly), we included only data from experimental sessions whose objective 2AFC hit rate increased with target contrast, and increased with reported visibility. If these criteria were met, we treated visibility levels where hit rate was near chance (50%) as “invisible faces” (or “visible Mondrians”), and higher visibility levels as “visible faces.” For all included CFS subjects, a division of trials into groups with visibility ratings of 4 (clearly visible) or 3 (mostly visible) and ratings of 2 (nearly invisible) or 1 (complete guess) satisfied these criteria. Summary measures of CFS performance for the included subjects are shown in Figure 4(left).

In the BM paradigm, a target face stimulus, whose emotional expression was either happy, angry, or neutral, was briefly flashed (one frame, ∼13 ms) at one of four visual field locations (upper-left, upper-right, lower-left, lower-right), which was immediately replaced by a gray blank screen. The same display settings as the CFS experiment, except for the mirrors were used. The face was placed within an oval shaped mask in the 1/f noise. The other three quadrants contained 1/f noise only. After a variable stimulus onset asynchrony (SOA), the stimulus array that included the target face was replaced with 1/f noise in all quadrants for 200 ms. SOAs varied from 13–213 ms. When the mask followed the target with a short SOA, the face was often rendered invisible. After each trial, subjects indicated the location and the emotion of the target face with two button presses. In absence of an explicit visibility judgment, we coded trials where subjects responded correctly for both location and emotion as visible, which can happen only once in twelve random guesses. We also treated trials where subjects incorrectly identified the location as invisible. Trials where the subject correctly identified the location but not the emotion were excluded from analysis for this study. We did not include the face emotion as an independent variable in any analyses for this study. Summary measures of BM performance are shown in Figure 4(Right).

In the unmasked paradigms (UNM), stimuli were presented on a continuous cycle while subjects fixated the center of the display. Stimuli included upright faces, upside-down faces, houses, line drawings of tools, and Mondrian patterns used in CFS (but not flickering). Subjects indicated either the change of the fixation cross color (“fixation task”) or, in separate experimental blocks, the repeat of stimulus category from trial to trial (“one back task”). Since most trials did not require a response, UNM blocks were included for analysis if the subject made responses during the task, but no accuracy threshold was imposed. For the analyses in this article, we did not distinguish between the fixation and one-back tasks.

Of these three tasks, only the CFS included a metacognitive judgment. Within a subset of CFS trials, subjects received physically identical visual input, yet they consciously experienced seeing the face target only on some of the trials. By dissociating stimulus parameters from conscious perception, this type of paradigm has been successfully used to isolate the neural correlates of consciousness (NCC; Koch, 2004). We include the other tasks as complementary to the CFS task to test the generality of our claims across different tasks. With regard to our classification of BM trials: nonconscious emotion discrimination has been reported in blindsight (Pegna et al., 2005) and in healthy subjects (Jolij and Lamme, 2005). However, we required correct discrimination of both location and emotion of the face targets, reducing the likelihood of including trials with a perceptually invisible face. In the UNM task, we presented the stimuli for a relatively long duration (500 ms) at fixation with no masks, no misdirection of expectation, and no attention demanding tasks. In such a situation, target perception is extremely unlikely to be nonconscious.

### Computing Φ*

Φ* is a measure of integrated information in a candidate subsystem *X*, derived in the following sequence of computations (*I–III*). For detailed mathematical derivation of Φ* (Oizumi et al., 2016a). A Matlab toolbox for computing Φ* may be found at https://figshare.com/articles/phi_toolbox_zip/3203326 (RRID:SCR_015549).

*I.* The state of an *n*-channel subsystem at time *t*, which we denote as *X(t)*, is an *n*-dimensional vector, where its *i* ^th^ dimension specifies the bipolar re-referenced voltage for the *i* ^th^ channel. To estimate the uncertainty about the states of the mechanism, we employ the concept of entropy, under an assumption of Gaussian distribution of these states (Barrett and Seth, 2011; Oizumi et al., 2016a):

(1) $$H(X)=\frac{1}{2}\log\left(\left|\Sigma (X)\right|\right)+\frac{1}{2}n\log(2\pi e),$$

where Σ(*X*) is the covariance matrix of X, estimated over the time interval [t-T/2, t + T/2]. T = 200 ms throughout the article; we found 200 ms to be a realistic range of the temporal window for conscious perception and a good compromise between better temporal specificity and better estimation of the covariances. The [*i,j*]*^th^* element of Σ(*X*) is the covariance between channel *i* and *j* of X over the time interval. |Σ(*X*)| is the determinant of Σ(*X*), known as the generalized variance (Barrett et al., 2010), as it describes the *n-*dimensional spread of instantaneous states of *X_t_*. The more different states *X* takes over the time interval, the more uncertain we are about its state at any time *t* within the interval.

*II.* Next, we estimate reduction in uncertainty about the mechanism's states at *t* (=*X_t_*) given its past states (=X_t-*τ*_, *τ* > 0) using the concept of mutual information *I*:

(2) $$I(X_{t};X_{t-\tau })=H(X_{t})-H(X_{t}|X_{t-\tau }),$$

where *H*(*X_t_|X_t-τ_*) is the conditional entropy of the mechanism *X* across the delay *τ.* Under the Gaussian assumption, the conditional entropy is given by

(3) $$H(X_{t}|X_{t-\tau })=\frac{1}{2}\log(|\Sigma (X_{t}|X_{t-\tau })|)+\frac{1}{2}n\;\log(2\pi e).$$

The covariance matrix of the conditional distribution, Σ(*X_t_*|*X_t-τ_*), is

(4) $$\Sigma (X_{t}|X_{t-\tau })=\Sigma (X_{t})-\Sigma (X_{t},X_{t-\tau })\Sigma {\left(X_{t-\tau }\right)}^{-1}\Sigma {\left(X_{t},X_{t-\tau }\right)}^{T},$$

where Σ(*X_t_*,*X_t-τ_*) is the cross covariance matrix between *X_t_* and *X_t-τ_*, whose element Σ(*X_t_*,*X_t-τ_*)*_i,j_* is given by covariance between i-th element of *X_t_* and j-th element of *X_t-τ_*.

The way we use mutual information here is similar to predictive information or auto-mutual information (Brenner et al., 2000; Gómez et al., 2007; Julitta et al., 2011). *I*(*X_t_; X_t-τ_*) is a measure of the information that current states have about their own past states.

*III.* Integrated information Φ* over the subsystem *X* is information that cannot be partitioned into independent parts of *X* (For simplicity, we remove t and t-*τ* from X for the explanation of Φ* here). To identify integrated information in a subsystem, we estimate the portion of the mutual information that can be isolated in parts of *X*. The process consists of first defining the parts of *X* (a “partition”) and then estimating the total mutual information under the assumption that the parts of a subsystem are independent. An estimate of “disconnected *I*” is called mismatched information and denoted as I* (Oizumi et al., 2016a; Oizumi et al., 2010).

We compute I* for every possible partition of *X*. As an example, if *X* is made up of four ECoG channels, there are 14 possible partitions of the subsystem (e.g., {a|bcd}, {ab|cd}, {ab|c|d}, {a|b|c|d}, etc.), excluding the “trivial partition” where all *n* elements are together in a single part (e.g., {abcd}). For each partition, we obtain Φ* = I – I*. We select the partition *P* that minimizes the normalized Φ* value, as defined previously (Balduzzi and Tononi, 2008):

(5) $$N_{P}=\left(m-1\right)\times \underset{k}{min}\left\{H\left(M^{k}\right)\right\}$$

(6) $$MIP=\underset{P}{\mathrm{argmin}}\left\{\frac{\Phi_{P}^{*}}{N_{P}}\right\},$$

where m is the number of partitions and *M^k^* is the *k^th^* part of the system *X*. The normalization term *N_P_* counterbalances inevitable asymmetries introduced by computing Φ* across variable numbers of partitions of unequal size (Balduzzi and Tononi, 2008). The partition that minimizes normalized Φ* is called the minimum information partition (MIP). The MIP reveals the weakest link between the parts of *X*. The integrated information of the subsystem is defined across the MIP as $\Phi_{MIP}^{*}=I-I_{MIP}^{*}$ (throughout this article, Φ* refers to $\Phi_{MIP}^{*}$).

For stable estimation of covariance and cross-covariance, we used a shrinkage approach (Schäfer and Strimmer, 2005). By computing covariance and cross-covariance matrices separately for each trial and averaging these before computing the entropy, we estimated Φ* for bins of trial data. For the classification analyses, bins consisted of three trials each (randomly selected from a given percept/stimulus category); for the exemplar Φ*-pattern illustrations shown in the Results section, all trials belonging to a particular percept/stimulus category were used to produce averaged covariance and cross-covariance matrices.

### The Φ*-pattern

To mirror the nested, multiorder structure of perceptual experience, we measured Φ* for every subsystem within a selected system of ECoG channels. For a system of four channels (ABCD), there are 11 such subsystems: ABCD, ABC, ABD, ACD, BCD, AB, AC, AD, BC, BD, and CD. The resulting set of Φ* values describes a pattern of overlapping subsystems that may or may not integrate information within the system. We refer to this set of integrated information as the Φ*-pattern. A structure based on the same composition of subsystems can be obtained using mutual information (I) or entropy (H). I and H sum linearly as subsystems are combined unlike integrated information, which may decrease as the size of the subsystem increases. I/H patterns were used as comparison for Φ*-pattern in statistical analyses (in Results, section ‘Classifying Conscious Contents’).

### Channel set selection procedure

We reasoned that where a stimulus evokes information integration, the pattern of the integration should identify the content of a conscious percept, especially in visual areas. To test this hypothesis, we conducted the following procedure for each specific experimental setting (a particular stimulus task, as viewed through a particular brain region of electrode implantation, either ventral or lateral regions, in a particular subject). The “max evoked Φ*” set of channels $\hat{X}$ is the subsystem and time lag (τ) where the largest average Φ* was evoked (averaged over all three-trial bins for all stimulus/percept categories):

(7) {X^, τ}=argminX, τ(Φ^*(X, τ)),

where Φ^*i was the largest average *evoked* Φ* for the i^th^ subsystem (i = 1, 2, …, 11) within a channel set *X* for a particular τ:

(8) Φ^*(X,τ)=maxi(Φ¯*i(X,τ)),,

and the mean evoked ${\bar{\Phi }}^{*}{}_{i}$ was the difference between the mean post-stimulus and mean pre-stimulus Φ* for the i^th^ subsystem for a particular τ:

(9) $${\overset{¯}{\Phi }}^{*}{}_{i}\left(X,\tau \right)=-\sum_{t=-300}^{-100}\Phi_{i}^{*}(t)+\sum_{t=100}^{300}\Phi_{i}^{*}(t).$$

In Equation 9, *t* is the center of a 200-ms interval of ECoG data, and the Φ* estimates are averaged over all three-trial bins for all stimulus/percept categories. We evaluated Φ*-patterns at each searchlight, and for each of four τ values (1.5, 3, 6, and 12 ms). Candidate channel sets included all positions of a rectangular searchlight in each subject’s lateral and ventral electrode arrays (a square searchlight for lateral arrays, and a slanted rhombus window for the ventral arrays). Most searchlight locations shared some two-channel subsystems with other overlapping locations, allowing some flexibility in the subsystem membership of the sampled channel set. If the highest evoked Φ* value was located a subsystem that belongs to more than one channel sets, the tie was resolved by the magnitude of Φ* of the subsystem that achieved next highest evoked Φ* within the competing channel sets.

**Figure 5.**
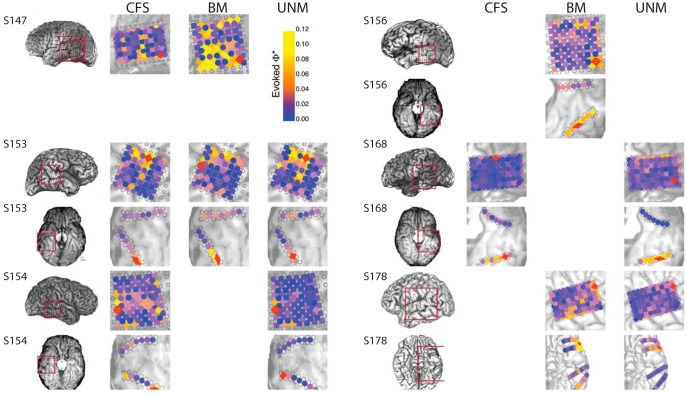
Max evoked Φ* searchlight and ROI selection. For each of 22 conditions (combinations of six subjects with ventral and/or lateral electrode implantation, and three stimulus paradigms, as represented by the rows and columns of the figure), we computed the average Φ*-pattern (over all experiment trials) for every four-channel system (square and rhombuses where each vertex is adjacent to the next), at each of four τ values (1.5, 3, 6, and 12 ms). As an ROI for further analysis, we chose one channel set that contained the subsystem with the highest Φ* regardless of stimulus/percept (see Materials and Methods for details). The selected regions for classification analysis (Figs. 9, 11) are marked in red. Estimated anatomic region of the max evoked-Φ* channel set are given in Table 1. Marker colors encode the magnitude of the maximal evoked Φ* at the centroid of each system (values above 0.1 are all given the color yellow).

**Figure 6.**
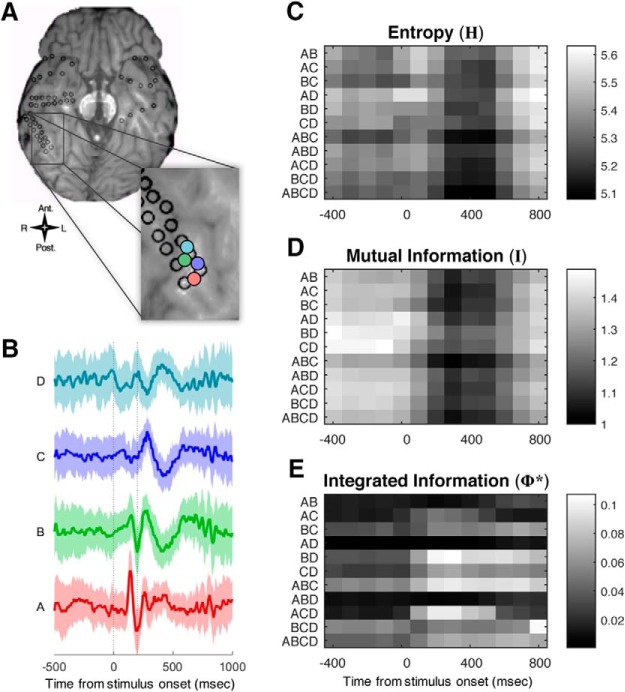
Measuring the integrated information pattern in ECoG data. ***A***, ECoG recording. Black rings mark the location of electrodes; we used bipolar re-referenced channels between each local pair of electrodes. Four of these are marked in color. ***B***, Means (thick lines) and SDs (shades) of the field potentials for the four channels marked in ***A***, from 500 ms before to 1000 ms after a face stimulus onset. Here, we included intervals over 45 trials in the CFS experiment where the high-contrast face target was reported as highly visible by Subject 153. ***C–E***, Time courses of the entropy *H* (***C***), mutual information *I* (***D***), and integrated information Φ* (***E***) for each of 11 subsystems. Each subsystem is a subset of the channels in the system ABCD. Values were estimated over a time window *T* = 200 ms and with time lag τ = 3 ms. Entropy and the mutual information are proportional to the number of channels, so we plot values in ***C***, ***D*** per channel for each subsystem, to emphasize the dynamics over all subsystems. The dynamics of Φ* are highly idiosyncratic. Note the increase in Φ* for subsystems BD and ACD, after the stimulus onset.

**Figure 7.**
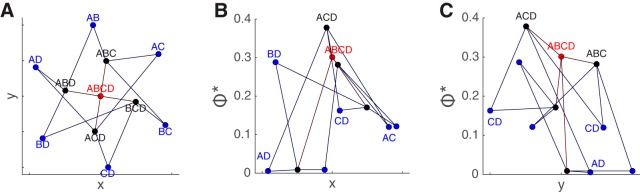
Integrated information pattern presented on a Hasse graph. See also Video 1. ***A***, The graph for all subsystems in the system ABCD as evaluated in Figure 6 (the same visible face trials in the CFS task) in the interval 200–400 ms. The *x* and *y* coordinates here are assigned for visualization purposes. Each node in the graph is one of the 11 subsystems in the system ABCD. The edges of the graph represent addition or subtraction of a single channel from a subsystem. The color of each node represents the number of channels in subsystem: blue nodes are two-channel subsystems, black nodes are three-channel subsystems, and the red node is the “top” four-channel subsystem**. *B***, The same graph viewed along the *y*-axis, with the magnitude of Φ* represented on the vertical (*z*) axis. Three-channel subsystem ACD labeled in black attained the highest Φ*. Adding or subtracting any channel to ACD only reduces Φ*. All the other subsystems in this system integrate less information than ACD, including the “enveloping” system ABCD. ***C***, The same graph viewed along the *x*-axis.

**Figure 8.**
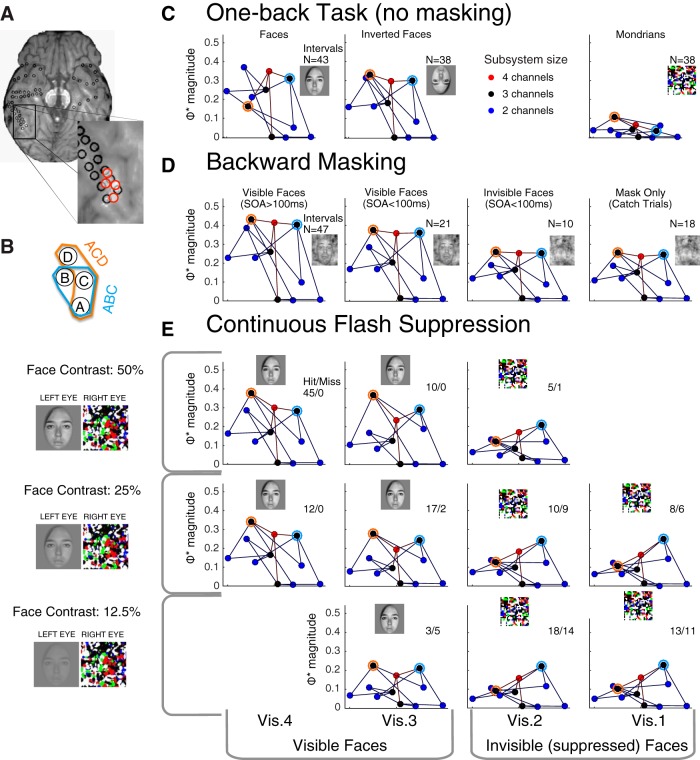
Patterns of integrated information corresponding to conscious perception of a face, generalizing across three completely different tasks and stimuli. ***A***, S153’s right fusiform gyrus. ***B***, Closeup of the channel configuration. Two prominent subsystems, ABC and ACD, are marked by the blue and orange circles in the following graphs (***C–E***). ***C–E***, Φ*-pattern graphs in the interval 200–400 ms after stimulus onset in multiple percept/stimulus conditions in three stimulus paradigms. Marker colors mean the same thing as in Figure 7. The same channel system in subject 153 is analyzed here as in Figures 6, 7. ***C***, Unmasked stimulus paradigm: Φ*-patterns for trials with the unmasked stimuli in the one-back experiment: faces, inverted faces, and Mondrians. ***D***, BM paradigm: Φ*-patterns generated in the four trial types in the BM experiment: visible face trials here are those where the subject correctly localized (4AFC) and identified emotion (3AFC) of the masked face, shown separately for long and short SOA trials; invisible face trials are those short SOA trials where the subject was incorrect for localization; and mask only trials were catch trials where no faces were presented. ***E***, CFS paradigm: Φ*-patterns from the interval that contained the face in the CFS experiment (Fig. 3). Φ*-patterns from the interval that did not contain the face were very similar to the pattern with visibility 1 or 2 in the face-present interval (data not shown). Within each row, the physical stimulus contrast was identical. Columns indicate the reported visibility of the face target. Hit/miss ratios are shown in each panel. The inset images roughly depict what was perceived in the corresponding intervals. To compute the Φ*-pattern in each panel, we used the number of trials available in the condition (N or Hit + Miss), as indicated by the inset numbers. Video 2 compares visible face trials in CFS and BM with invisible trials in CFS in a 3D rendering.

The max evoked Φ* set $\hat{X}$ for each condition was used for the classification analysis and included in the ANOVA and AUC time course (Fig. 11*A*) described in the results. The searchlight location of the largest average evoked ${\hat{\Phi }}^{*}{}_{i}$, for each condition, is plotted in Figure 5, with its anatomic location listed in the last column of Table 1. The exemplar ROI featured in Figures 6–8 was the max evoked Φ* set for S153’s ventral BM condition (the highest overall evoked Φ* over three stimulus paradigms for this subject), a set of channels over the right fusiform gyrus. The max evoked Φ* set for the CFS condition was next to the system depicted in Figure 5.

### Unsupervised clustering for assessing the similarity of informational patterns

We used unsupervised classification for all available subjects within each stimulus paradigm and within recording locations (either lateral or ventral temporal surface), comparing classification performance across the three types of information pattern (Φ*, I, and H). As input features to the clustering, we reduced the dimensionality of the input features into the first 4 multidimensional scaling coordinates derived from (Pearson) correlation-distance matrices (Kriegeskorte et al., 2008), and used the nearest-neighbor algorithm for percept classification.

Measurement of conscious percept classification began with defining categories for each experimental stimulus interval. For CFS, we performed two analyses. In Figure 11*C*, we used only the intervals that contained mid-contrast target faces (so that visual input was the same across all trials) then categorized each trial according to the reported percept in each trial as either visible face (visibility 3 or 4) or visible Mondrian (visibility 1 or 2; Fig. 3). For Figure 11*B*, we used all available intervals for CFS; the percept categories were either visible face (face-present intervals with visibility 3 or 4 regardless of the contrast of the face) or visible Mondrian (face-present intervals with visibility 1 or 2 as well as face-absent intervals). For BM, they were either visible face (trials with correct discrimination in both location and emotion regardless of SOAs, where random responses would result in only 8.3% accuracy) and Visible Noise (trials with incorrect location discrimination regardless of SOAs as well as catch trials where no face was presented). For UNM stimuli/percepts were either faces (including upright and inverted faces) or nonfaces.

We assessed the clustering with a cross-validation procedure. Cross validation removed any possible bias due to unequal number of trials due to percept. First, using 70% of randomly sampled trials within each percept category as a training set, we determined the center of gravity for each category. Second, using the remaining 30% of trials as a test set, we obtained a series of hit and false alarm rates by gradually changing a criterion distance from the category center. We varied the criterion radius for each category from the mean estimated from the training set, counting the number of same-category bins within the radius as “hit” and other-category bins as “false alarm.” By smoothly varying the radius from 0 to infinity, the proportion of both Hit and False Alarm changes from 0–1, yielding a receiver-operating characteristic (ROC) curve for each class. We averaged the area under the ROC curve (AUC) over all categories as the measure of the classification accuracy. The procedure was repeated 20 times, with random resampling of train/test bins. For statistical analysis, each AUC value was converted into a zAUC score through the inverse cumulative normal distribution, which removes the 0–1 bounds of the AUC value and is more appropriate to ANOVA.

## Results

### The structure of integrated information

We measured information patterns (Φ*, I, and H) at every location of a four-channel searchlight, in each of six subjects, over 22 conditions (different tasks and electrode settings; Fig. 5) for a range of τ (time lag) values from 1.5–12 ms. All computations were performed over 200-ms time windows spaced 100 ms apart over a period from 400 ms before stimulus onset to 1000 ms after onset. To select regions of interest, we focused on maxima in evoked Φ*, i.e., the searchlight locations with maximal increase in Φ* after stimulus onset, regardless of the stimulus category.

We first present a detailed look of an integrated information pattern in a single subject (S153) for the CFS task, in a group of channels located over the right fusiform gyrus, a region that has a known association with conscious perception of faces (Tong et al., 1998; Puce, Allison, and McCarthy, 1999; Parvizi et al., 2012; Rangarajan et al., 2014). The process is illustrated in Figure 6 for a system of four channels (Fig. 6*A*). These four channels (with τ = 3 ms) contained the highest evoked Φ* of any searchlight location in this subject (Fig. 5, S153). Figure 6*B* plots the average time course of raw bipolar re-referenced voltages (no baseline correction) during high-contrast face intervals in the CFS task, where subjects felt faces were highly visible (visibility ratings of 4). We computed Φ* for every subset of at least two channels from this system: for a system of 4 channels this yields 11 subsystems: six two-channel subsystems, four three-channel subsystems, and a single four-channel subsystem. Figure 6*C–E* shows the time courses of the quantities that underlie Φ*, computed for each of 11 subsystems. The most general quantity is the entropy *H* (Fig. 6*C*). From the entropy *H*, we subtract the conditional entropy, yielding the mutual information *I* (Fig. 6*D*). The integrated information is the difference between the mutual information and the sum of the mutual information of its parts: Φ* = *I* - I* (evaluated at the MIP of the subsystem; Fig. 6*E*).

Most subsystems show a similar time course for *H* and *I* (in the example system of Figure 6 as well as other observed systems), with entropy and mutual information decreasing a few hundred milliseconds after stimulus onset. In contrast, Φ* has an idiosyncratic time course, strongly dependent on the specific subsystem. For most subsystems, Φ* remains near zero regardless of the visual stimulus; for others, it may start high and drop after a particular stimulus is presented (Fig. 6*E*, subsystem BCD); and for other subsystems, Φ* starts low and increases after stimulus presentation (Fig. 6*E*, subsystems BD and ACD).

### Φ*-patterns as graphs

The Φ*-pattern is not just a vector of integrated information values; each value has a necessary relation to some of the others, in the form of an inclusion or intersection relation. This property of the Φ*-pattern distinguishes it from more orthodox measures that aim to reduce neural activity to independent components: the components of the Φ*-pattern are by design not independent. The inclusion relations in a Φ*-pattern can be illustrated in the manner of a Hasse graph (Fig. 7*A*). In the graph, we place the highest four-channel subsystem ABCD at the origin (the red colored node), surrounded by the three-channel subsystems in black, which are further surrounded by the two-channel subsystems in blue. The edges represent adding or subtracting one channel to a subsystem. For example, three-channel subsystem ACD is connected to subsystems AC, CD, AD via edges as well as ABCD. The pattern is more easily appreciated in a 3D rendering (Video 1).

Video 1. Four-channel Φ*-pattern (from Figs. 6, 7), rotated through 3D to clearly illustrate its construction. The *x*-*y* coordinates are arranged to illustrate the layout of the Hasse graph that connects all the subsystems. The *z*-coordinate is the magnitude of Φ* for each subsystem. This pattern is generated by a system of four channels over subject 153’s posterior fusiform gyrus. 10.1523/ENEURO.0085-17.2017.video.1

The four-channel Φ*-pattern in Figure 7 illustrates an important property of Φ* that distinguishes it from H and I: nonlinearity and nonmonotonicity with respect to the size of the subsystem. Here the three-channel subsystem ACD attains the highest Φ* of all 11 possible subsystems. The graph illustrates how subtracting from or adding to a subsystem can reduce the integrated information. The Φ* of ABCD is less than that of ACD because there is a relatively weak interaction between B and ACD. In this case, the MIP for ABCD is between B and ACD.

Figure 8 shows Φ*-patterns obtained in three separate experiments: CFS, BM, and the unmasked one-back task (For comparisons of these patterns, see Video 2 to view Φ*-patterns in a 3D perspective). Φ*-patterns in the first two columns are constructed from trials where the subject clearly perceived a face (94 trials with high visibility ratings in CFS, with hit/miss counts identified in the inset text, 68 trials with correct in both location and emotion discriminations in BM, and 81 trials of unmasked upright or inverted faces in the one-back task), whereas the last two columns are not (95 low visibility rating CFS trials, 28 incorrect location discrimination BM trials, and 38 Mondrian trials from the one-back task). In this example, Φ*-patterns in trials with clearer face percepts (the left columns) have have greater magnitude and generally build toward the ACD subsystem (outlined in orange); while nonface percepts have lesser magnitude and, at least in the CFS conditions, build toward the ABC subsystem (outlined in blue) – in fact, the integration of the ABC subsystem seems relatively invariant to percept condition. The shapes of the graphs are similar within the visible-face columns, implying that Φ*-patterns generalize across conscious perception of faces in the three different stimulus/task contexts. This is consistent with a proposed isomorphism between conscious perception of faces and the Φ* structures, which are invariant to task or stimulus details (Aru et al., 2012; de Graaf et al., 2012).

Video 2. Four-channel Φ*-patterns for visible BM faces (left pattern), visible CFS faces (middle pattern), and invisible CFS faces or Mondrians (right pattern). Similar patterns from the same subject 153 are shown in Figure 8. 10.1523/ENEURO.0085-17.2017.video.2

**Figure 9.**
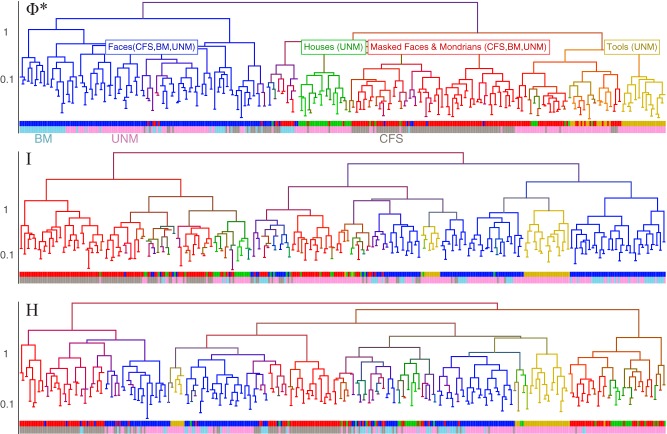
Pattern clustering for the channels featured in Figures 6-8 in subject 153, across 364 bins of three trials each. Three dendrograms are illustrated based on the patterns of Φ* (top), mutual information I (middle), and entropy H (bottom). The dendrograms represent relative (Euclidean) distance between patterns. Four coarsely defined percept categories are conscious perception of faces (blue), masking stimuli (red), houses (green), and “tools” (yellow). The red category, conscious perception of masking stimuli, corresponds to the two right columns in Figure 8, and it consists of UNM Mondrian trials, BM invisible trials (location discrimination incorrect) and catch trials (no face presented), CFS invisible face-present intervals (visibility 1 or 2), as well as CFS face-absent intervals (Fig. 3). Thus, we analyzed all available trials in BM regardless of SOAs and CFS regardless of presence or absence of the face as well as all face contrasts. Below each dendrogram are two bands of colors: the upper band identifies the percept category for each dendrogram leaf (trial bin), and the lower band identifies the stimulus paradigm: CFS (dark gray), BM (light blue), and UNM (light magenta).

The patterns in Figures 7, 8 were obtained by averaging covariance matrices over variable numbers of trials within each stimulus/percept category (seen-faces, masked-faces, etc.; between 6 and 47 trials for the different stimulus/percept groups). To illustrate the same mapping at a finer grain, we computed Φ*-patterns for bins of three trials each (with identical stimulus/percept categories contributing to each bin), yielding 364 Φ*-patterns generated over the different stimulus paradigms (for the same subsystem in Subject 153). The Φ*-patterns shown in Figure 8 were derived by combining all available trials for each stimulus/percept condition; if we break the data down and derive a Φ*-pattern for just a few trials’ worth of data, the mapping to stimulus/percept still holds: Each dendrogram leaf in Figure 9 corresponds to a Φ*-pattern for a bin of three stimulus/percept-matched trials. Φ*-patterns corresponding to visible face percepts cluster together and separately from Φ*-patterns generated during other percepts. For comparison, we conducted the same analysis for patterns of mutual information and entropy (I and H; middle and lower panels, respectively) across the same set of subsystems as in the Φ*-pattern; these appear to yield poorer clustering. This suggests that refining this particular neural system’s activity to a pattern of integrated information results in a closer structural match to conscious perception of faces across different task contexts, and reasonable classification of other percept categories as well. However, these “by eye” analyses of the mapping between Φ*-patterns and conscious contents are merely suggestive; in the next section, we apply objective clustering analyses over a group of subjects.

### Classifying conscious contents with integrated information

To evaluate the mapping between percept and Φ*-pattern, we constructed matrices representing dissimilarity (the Pearson distance) of Φ*-patterns derived from bins of three stimulus/percept-matched trials. As a dimension-matched comparison, we also measured dissimilarity for entropy (*H*) and mutual information (*I*) patterns. We used these dissimilarities as the basis of a clustering analysis to determine how well the different types of information pattern mapped onto the known percept/stimulus condition for each bin of trials (see Materials and Methods for details). Importantly, there was no training step to optimize weights for this unsupervised classification: the pattern coordinates served directly as unweighted feature vectors. This is important because our motivation for this representation similarity analysis was not to find the most accurate decoder. Rather, we wanted to test if a hierarchy of integrated information might show direct isomorphic correspondence with conscious percepts (see Discussion).

**Figure 10.**
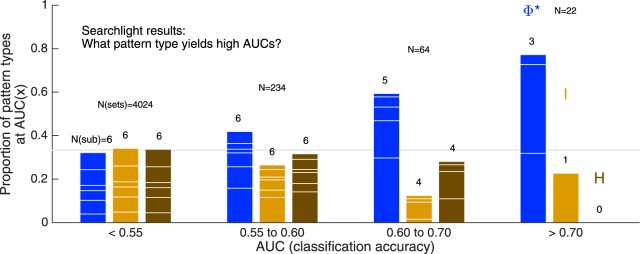
The likelihood of each pattern type given specific AUC ranges, over all searchlight locations, all stimulus paradigms, and all subjects. AUC is taken as the average between 200 and 400 ms after stimulus onset. Likelihood is the incidence of a pattern type at the given AUC, divided by the total number of sets yielding the given AUC (indicated by the *N* over each group of bars). Thus, the chance level is 0.33 (i.e., one of three pattern types), indicated by the horizontal gray line. Each column is subdivided according to the contribution of six subjects, the numbers over each column indicate the number of subjects contributing to each pattern type at that AUC range. This analysis does not rely on a particular channel selection strategy, and emphasizes that accurate matches with percepts (AUC > 0.6) are predominantly found with the Φ* pattern.

We obtained local Φ*, *I*, and *H* patterns at hundreds of “searchlight” systems in each subjects’ electrode arrays, with each pattern computed on a set of three percept/stimulus-matched trials’ worth of data (see Materials and Methods for details). For each experiment condition (subject/electrode array/task) we conducted the classification analysis separately at every searchlight location. Figure 10 summarizes the searchlight classification results, combining AUC scores over all experiment conditions by expressing the likelihood of each pattern type given a specified AUC range. In other words, if a searchlight system yielded a certain AUC, what is the probability that it was with an integrated information pattern, a mutual information pattern, or an entropy pattern? A high degree of pattern similarity to conscious percept is very rare (most searchlight locations yield chance performance), but when similarity *is* high Figure 10 shows that it is most likely to be with a Φ*-pattern.

**Figure 11.**
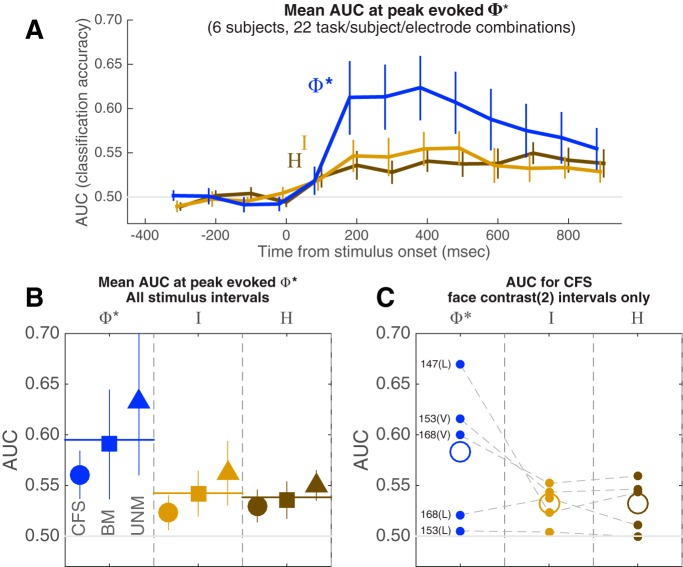
Percept classification accuracy for different pattern types. ***A***, ***B***, Trials were classified within each task. For UNM, they were classified as either faces or nonfaces; for BM, as visible versus invisible face trials (including catch trials as invisible faces); and for CFS, as visible face-present intervals versus all other intervals (including invisible and absent faces). ***A***, Average time course of pattern classification (AUC) over 22 conditions (combinations of six subjects, three possible stimulus paradigms, and either lateral or ventral electrode implantations), where the four-channel sets were selected based on their maximal evoked Φ*. Φ*-patterns classify percept category better than mutual information and entropy patterns (I, H). Error bars are the SEM. ***B***, Average AUC over the period between 100 and 600 ms after stimulus onset, in each of the three stimulus paradigms, for each pattern type. ***C***, Classification accuracy for CFS trials, restricted to middle contrast face-present trials (corresponding to the middle row of Fig. 8*E*) for each of three observers [two with both ventral (V) and lateral (L) electrodes]. In this analysis, all trials were identical in physical stimulus contrast; above-chance classification relies only on differentiation of perceptual state. As with the analysis in ***A***, ***B***, the Φ* structures match contents of consciousness better than H or I structures in each observer.

Next, we selected the searchlight system that generated the largest evoked Φ* regardless of stimulus/percept for each distinct experiment condition (as listed in Table 1). Note that using the same criterion, we selected the system featured in Figures 6-9 for S153; see Materials and Methods for details). Figure 11*A* shows the average AUC time course for these systems based on integrated information (Φ*), mutual information (I) and entropy (H); average classification is best with Φ*-patterns. Figure 11*B* shows the same result broken down by stimulus paradigm: integrated information outperforms in both masked and unmasked stimulus paradigms.

Importantly, the same result is obtained when classification is performed only on stimulus-identical CFS face-present intervals (Fig. 11*C*). During these intervals, the physical input to the brain was identical: mid-contrast faces paired with Mondrian masks, and the only variable was the subjective visibility of the face; the best classification of subjective visibility was obtained with the Φ*-patterns, in each of three observers (a fourth CFS observer, S154, did not supply enough data to compute AUC for any single contrast level).

We used the poststimulus-onset zAUC scores (see Materials and Methods) over all conditions (combinations of six subjects, three tasks, and two types of electrode installation; see Materials and Methods), with pattern type, subject ID, and task as fixed factors and post-stimulus time point as a covariate for an ANOVA. There was a main effect of pattern type (*F*_(2425)_ = 15.332, *p* < 0.001), with significant (Bonferroni-corrected) post-hoc pairwise differences between zAUC derived from Φ* versus *I* patterns (*p* < 0.001), and Φ* versus *H* patterns (*p* < 0.001), but not between *I* and *H* patterns (these comparisons reflect differences between the poststimulus time courses in Fig. 11*A*). There were also significant main effects of subject ID (*F*_(5425)_ = 47.802, *p* < 0.001) and task (*F*_(2425)_ = 14.866, *p* < 0.001). Except for the pattern-type-by-task interaction, all other factorial interactions were significant, likely reflecting the large heterogeneity of the data conditions.

**Figure 12.**
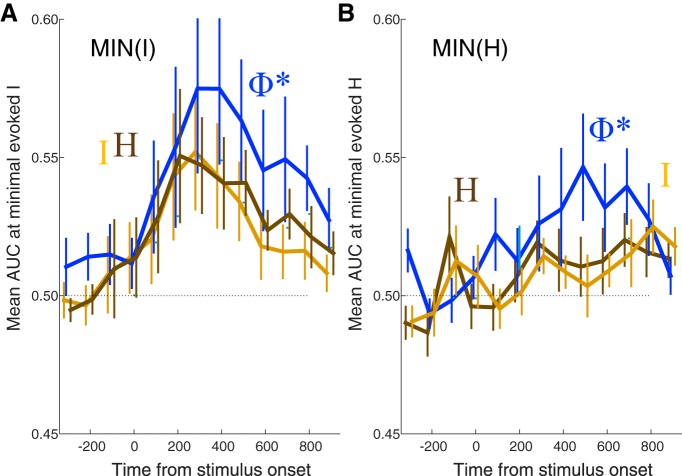
***A***, ***B***, Selecting systems based on a “minimal mutual information or entropy” criterion, rather than a max evoked Φ* criterion, still picks out systems where Φ* structures match with conscious percept better than other structures, although peak AUC is not as high as when the criterion is max evoked Φ* (as in Fig. 11).

In the preceding analysis, we simply reasoned that where information is integrated (or causal interactions are evoked) in response to a stimulus, its pattern should map closely to the reported experience. Although Figure 11 shows the advantage of Φ* over I or H in most systems in the ventral and lateral cortex, using the above procedure to select ROIs for statistical analysis may give AUC(Φ*) an unfair advantage over AUC(*I*) and AUC(*H*). In fact, AUC(Φ*) is correlated with evoked Φ*. To evaluate this problem, we also selected channel sets based on the minimum or maximum evoked *I* and *H.* Regardless of the selection criteria, however, the highest AUC was obtained with Φ*-patterns (Fig. 12), although the overall level of classification tends to be poorer than when the criterion is based directly on Φ*.

## Discussion

We have presented a novel analysis which allowed us to evaluate the degree of similarity between integrated information patterns in neural activity and specific conscious experiences. Some theories of consciousness, most prominently the IIT (Tononi, 2004), predict that conscious experience should closely match with a hierarchical pattern of causal neural interactions. Although our investigation of this hypothesis cannot be posed as a test of the IIT per se (see below), we did find that the integrated information pattern in human cortex closely matched with psychophysically-established perceptual contents. This finding generalized across stimulus paradigms and included conditions where physical stimuli were dissociated from perceptual experience. Furthermore, Φ*-patterns map more closely to perceptual states than do entropy and mutual information patterns, despite their overlapping derivation.

**Figure 13.**
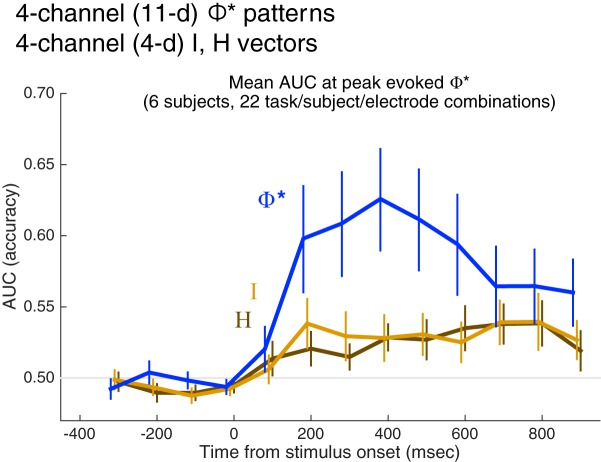
To isolate the contribution of self-interactions, we repeated the main analysis described in Figure 11*A*, but we used only H and I derived from each channel (i.e., {H(A), H(B), H(C), H(D)}, etc.). Using these 4D patterns, instead of 11D pattern, we performed the same analysis, but the results were very poor, consistent with a critical role of cross-channel causal interactions.

The advantage of Φ* over entropy and mutual information is likely due to Φ*’s isolation of integration. Entropy quantifies only the instantaneous uncertainty of the neural states, understandable as equal-time interactions (Oizumi et al., 2016b; Fig. 2*A*). Mutual information quantifies causal (time-lagged) interactions in these distributions, i.e., how past states affect future states, including both within and between channels (Fig. 2*B*). Φ* also quantifies time-lagged interactions but only those that tie groups of channels together (Fig. 2*C*). Thus, our results can be summarized as showing that neither the pattern of equal-time interactions (H) nor of time-lagged self-interactions (I) resemble patterns of conscious percepts as well as causal cross-interactions (Φ*). This suggests a critical role of causal interactions across the system of neuronal activity for understanding how conscious phenomenology corresponds to neural systems. Indeed, when we applied our analyses to patterns of self-interactions (i.e., patterns of single-channel based entropy and mutual information; Fig. 13), the pattern did not correspond to conscious percepts at all.

Another way to understand the advantage of Φ* is as noise reduction: each step in the derivation of Φ* involves not the construction of new information from the neural data, but “deletion” of noise, thus improving the performance of the clustering algorithm. When mutual information is computed from entropy, it is by subtracting the part of the system noise that is not carried across the time lag τ; when integrated information is computed from mutual information, it is by subtracting the part of the system noise that is only within parts of the system. Derivation of Φ* can then be seen as reduction of the system noise to only those portions that persist across the time lag, and across the parts of the system. This residual maps closely onto the quality of a conscious percept.

In the notable case of subject 153 who provided a large number of trials in the CFS and other experiments, we observed a similar Φ*-pattern generated in different stimulus paradigms when the subject experienced a “face” percept. The elements generating the Φ*-pattern, channels of neural activity in the right fusiform gyrus, have long been associated with face percepts on the basis of patterns of responsivity and perceptual consequence of electrical stimulation of the area (Tong et al., 1998; Haxby et al., 2000; Grill-Spector et al., 2004; Parvizi et al., 2012). Different populations of neurons in this region are known to encode different dimensions of face structure (Freiwald et al., 2009), but for these dimensions to be experienced as parts of a whole there must be some higher-order integration across multiple populations. Here we have demonstrated that when a face is seen, activity across multiple populations in this area is irreducibly integrated across multiple hierarchical levels to result in a unique pattern of integrated information, and that this integration is correlated with conscious percept on a trial-by-trial basis. Generalizing this finding, we have demonstrated in a number of subjects, stimulus paradigms, and cortical regions, that how the information is integrated (the Φ*-pattern) indeed reflects the nature of the percept evoked by a stimulus.

One caveat typical of ECoG recording in human patients is that our data are influenced by large variability in electrode implantation locations (Fig. 5) which likely contributed to the variance in the quality of classification across condition. Furthermore, while we postulate that the different recorded populations contributing to “face percept” Φ*-patterns are involved in encoding different dimensions of complex face experiences (see the next section on Interpretation), our experiments were not designed to investigate these details. A more theoretical caveat is that while we are clearly limited in measuring integrated information across neural populations as measured by ECoG electrodes, this does not mean that the true underlying integrated information structure involves populations as basic units; the integrated information structure corresponding to conscious experience may exist at a much finer scale such as just a few neurons or even more local physical structure (Barrett, 2014; Hoel et al., 2016; Tononi et al., 2016). We must therefore be cautious in interpreting the meaning of Φ*-patterns obtained from such coarse recordings as ECoG electrodes which sum responses over many tens of thousands of neurons; accordingly, our procedure is not a direct test of IIT.

### Interpretation of integrated information patterns

In theory, the low-order Φ* values for local parts of the system would correspond to particular features or dimensions of a larger experience; the higher-order Φ* values would then correspond to the binding of these features. For example, suppose that connected but distinct neural populations encode the presence of particular facial features (an enormous simplification; Freiwald et al., 2009; Henriksson et al., 2015; Chang and Tsao, 2017), with one population encoding *eyes* (population E), another encoding *nose* (population N), another encoding *mouth* (population M), and another encoding a *group* of features (population G). When a face is seen, each population will be active, but the features must be somehow bound together in a whole to correspond to the holistic experience of a face composed of features; that is, there must be a physical substrate that reflects the integration of facial features. For such a system of neural populations, when a face is seen, activity in mouth might be moderately integrated with activity in nose (i.e., Φ*{M,N}>0), nose with eyes (Φ*{N,E}>0), all three features together (Φ*{M,N,E}>0), and any of these should be strongly integrated with the group population (Φ*{E,G}>0, Φ*{M,G}>0, etc.); meanwhile, mouth and eyes populations may not be integrated at all (i.e., Φ*{M,E}≈0). The Φ*-pattern generated by these populations during visual experience of a face would therefore have a particular structure isomorphic to the complex experience. While we have no way of ascribing particular functional roles to the ECoG channels in our dataset, this scenario suggests a plausible interpretation for Φ*-patterns.

The idea of an informational hierarchy having a close relation to the substrate of consciousness may seem familiar: computational theories (e.g., Marr, 1982) and more recently predictive coding theories (e.g., Hohwy, 2013) of the mind heavily rely on the notion of hierarchical substrates. In these schemes, a “high-level” neuron may receive inputs from “low-level” neurons, and feed back onto the lower level to adjust the sensitivity of its inputs. However, the neurons themselves cannot know their location within the hierarchy. Likewise, they cannot know which inputs are from high- or low-level neurons. The hierarchy is only there in the description of the system by the experimenters. IIT does not take on this kind of extrinsic viewpoint: the hierarchical structure of a system emerges intrinsically as the integration of the system’s parts. Thus, we did not assume any hierarchy of ECoG channels, but instead extracted a hierarchical pattern intrinsic to the activity across those channels. This is an important property afforded by IIT that does not seem to be obtained from hierarchical models of information processing in the brain.

Considered in this way, even detached from IIT, the notion of an intrinsic hierarchy of integrated neural structures may prove to be extremely fruitful for future neuroscience research. In fact, current perspectives on neural processing do seem to be moving toward high-dimensional descriptions of how connected brain systems accomplish the jobs of consciousness, perception, and cognition. Looking at synapse-level connectivity of large groups of single neurons (Reimann et al., 2017) have recently shown how neural systems can be understood in terms of their topological features, treating them as sets of overlapping groups of connected neurons. In another study, Lord et al. (2017) have described how a different set of methods to distinguish “integration” (the holistic property of a neural system) from “segregation” (the way the parts are differentiated from one another) can be used to understand psychiatric disorder and other aspects of whole-brain function.

### A novel approach toward finding the neuronal isomorphism of consciousness

As we emphasized in Results, we intentionally avoided “optimizing the classifier” by finding the best weights to achieve higher AUC. While such decoding analyses have their own advantages in the search for NCCs (as in the same data set presented in this paper; cf. Baroni et al., 2017), there is an important caveat in such an approach: the assumption of the decoder. Even if one can perfectly identify conscious perception based on patterns of neuronal activity, the interpretation of a vector of regression weights, for example, is entirely dependent on the presence of a decoder, usually a tool in the hands of the experimenter. What we learn from this kind of analysis is that the information necessary for consciousness is available in (e.g.) a certain brain area. But there is no clue as to what it should feel like, why should one vector of weights feel so different from another? A hierarchical structure of integrated information does not pose the same problem, because it is hypothesized a priori as the structure of experience, serving as a theoretical basis for the experiment.

Accordingly, our goal in this study was not to find the optimal classifier for these data; our goal was to test the prediction that an integrated information pattern in the brain corresponds to the contents of consciousness. Our approach is therefore distinct from the traditional “search for NCCs.” We aimed not to build a perfect classifier that presupposes an ideal homunculus who reads the encoded information, but to test if a hierarchy of causally connected and integrated information can possibly relate to contents of consciousness. Our results provide an affirmative answer.

### Summary

A conscious experience is intrinsic, existing for itself, not for some outside observer, Φ* measures how a system specifies its own state, not an external state; a conscious experience is integrated, irreducible, more than the sum of its parts, Φ* measures how the system as a whole specifies its own state, above and beyond its elements; and a conscious experience is compositional, just like the hierarchical Φ*-pattern. Based on these theoretical parallels, there is strong a priori reason to expect Φ*-patterns should closely correlate with perceptual experience. This type of theoretical background is lacking in many investigations of the NCC. In this regard, our study can be seen as a test of a theory, albeit limited and indirect as we acknowledge above, rather than the traditional search for NCCs.

In this study, we have tried to keep things simple, concentrating on four-channel systems, and have been limited by the (experimentally) incidental placement of recording sites. Our results should be seen as a first step in the direction of a new empirical research framework in consciousness science, where we assess the degree of structural similarity between conscious phenomenology and theoretical construct, with empirical data as a bridge between the two (Tsuchiya et al., 2016; Tsuchiya, 2017). Future studies may use data that is obtained explicitly for the purpose of extracting integrated information patterns, over larger and larger data windows. If there is indeed an isomorphism, which the current study can only suggest, then the structure of a conscious experience should be similar to the structure of the theoretical construct, pointing to a need for assessment of phenomenological structure alongside collection of neural evidence.

Looking further ahead, IIT would require that for a local Φ pattern to be a part of conscious experience as a whole, it must be a part of a much larger pattern extending across multiple cortical regions. Our procedure did not measure this superstructure, called a complex in IIT (Tononi et al., 2016), another reason that our procedure cannot be taken as a direct test of IIT. We see several other immediate avenues of development that should follow from our study; first, we would propose that new tests of the Φ-pattern concept should include conditions of constant stimulation under varying levels of consciousness (e.g., comparison of Φ-patterns during wakefulness and anesthesia), similar types of Φ-pattern should be observed during dream states as during wakefulness, but different types (with much lower Φ magnitude) should be observed during dreamless sleep or deep anesthesia.

With the methodological framework we provided here, it will be possible in the future to test a subset of the theory’s predictions, that hierarchies of integrated information are isomorphic to the structure of conscious contents, i.e., that the binding problem may be resolved by this approach. The Φ-pattern concept can be applied to other data types in other experimental manipulations combined with neural recording and stimulation, under various conscious states (e.g., awake vs anesthetized), approaching ever closer to establishing the link between the mental and the physical worlds.

## Synthesis

Reviewing Editor: Gustavo Deco, Universitat Pompeu Fabra Decisions are customarily a result of the Reviewing Editor and the peer reviewers coming together and discussing their recommendations until a consensus is reached. When revisions are invited, a fact-based synthesis statement explaining their decision and outlining what is needed to prepare a revision will be listed below. The following reviewer(s) agreed to reveal their identity: Phillip Alday.

Using ECoG data, the authors examine which information-theoretic measure -- entropy and mutual information from classical information theory and integration information from a more contemporary source -- best tracks “conscious” information processing in the brain as indexed by detection of target stimuli in various masking paradigms. They argue that the partitioning scheme inherent in integration information, which is designed to capture the hierarchical structure in complex signals, parallels the hierarchical structure of conscious experience. Therefore, they argue, the better decoding performance (model fit) of integrated information on their conscious information processing task(s) shows that integrated information best tracks the emergence of conscious information processing.

I find the performance of integrated information compared to more classical measures (entropy and mutual information) to be very interesting, especially as integrated information is a function of entropy of the subsets / partitions of the data. However, I am not convinced that the present manuscript addresses the central issue of its current formulation, namely the “significant role of locally integrated information for understanding the neural substrate of conscious experience” (from the abstract) or the “phenomenon occurring in brains that is critical for consciousness” (from the significance statement).

There seem to be two motivating ideas behind the claim that the authors are capturing (the emergence of) consciousness: (1) their dependent measure is “conscious awareness of the percept” based on behavioral performance in a detection task and (2) the parallel structure of integrated information to the structure of conscious perception. Let me address these in turn.

I'm not sure that detection performance is sufficient for consciousness. An amoeba can detect elements of its environment and produce an appropriate behavioral response without anything approaching a nervous system. Accurately assessing the facial expression as opposed to simple target detection does address some of this -- it would seem that facial expressions require something like consciousness but then again, non-human primates whose conscious nature is more difficult to assess are also capable of processing facial expressions. The difficulty here may very well lie in defining what consciousness is, which brings me to the other potential motivating idea, the parallel structure of integrated information to the structure of conscious perception.

The authors argue that conscious experience has an intrinsic, integrated, and hierarchical structure. However, this cannot be the defining quality of conscious experience because this type of structure is found everywhere. In neuroscience, this is found e.g. in Friston's predictive coding framework (which I would suggest that the author's examine for useful parallels and connections to integrated information), yet predictive coding is not a theory of consciousness -- one of the classic examples for predictive coding is the MMN, which can be elicited from coma patients.

(And to fully argue integrated information being isomorphic to conscious experience, the authors would need to demonstrate that its decoding performance is bad / worse than classical measures for unconscious states / behaviors. Maybe something demonstrating that integrated information processing tracks P300 related activity but not MMN?)

Again, I find the integrated information and its performance quite interesting, but I'm not sure the present paper demonstrates that it indexes conscious experience -- perhaps “perception” and maybe “conscious perception” but not “conscious experience”. My concrete suggestion would be to focus on an experimental validation of the integration information as capturing some aspect of perception and neural processing -- notice the lack of “conscious”. In the discussion, it would be appropriate to tie into other literature and note that the present data provide are compatible with (and perhaps provide converging evidence for) a theory of consciousness based on integrated information.

(I'm also curious as a thought experiment what would happen with this measure in blindsight patients, who can avoid obstacles despite having no / a very different conscious visual perception of them.)

I found the first half of the manuscript to be fairly well written. There is a fair amount of mathematics, which will be challenging to some readers, but the authors do a decent job in their exposition. The organization isn't always ideal, but this is not particularly problematic in the first part of the manuscript.

The writing really breaks down around the start of the Results section, where large portions seem to be nearly exactly duplicate previous paragraphs. The clarity of the mathematical exposition also drops at this point. The authors should re-work this section so that there is less repetition and more clarity and “flow”. This could also potentially shorten the manuscript a fair amount -- short is not always better, but short will generally increase your readership. As I think this needs a fair amount of revision, I won't list individual edits.

Statistical comment (in response to the eNeuro prompt “Is the statistical analysis correct?”):

==========================================================

The statistical analysis using an ANOVA over z-transformed ROC AUC may be technically correct (I'm not entirely sure). I'm assuming that the authors performed a Fisher z-transformation to avoid issues with boundedness inherent in the ROC AUC measure, but “z-transformed” (i.e. without “Fisher”) can also refer to the standardization/scaling procedure where data are expressed as standard deviations away from the mean. The latter transformation would not be appropriate. Moreover, the Fisher z-transform does not address the boundedness issue for ROC AUC because it is still bounded at zero. For correlations, which go from -1 to 1, this is not the case; but AUC only goes from 0 to 1.

Perhaps a simpler method than ROC AUC + Fisher transform would be to use d-prime.

I find explicit regression techniques to almost always be better than ANOVA procedures.

I'm also a bit concerned by the uneven distribution of trial numbers (e.g. in Fig 4) when calculating proportions. I realize this may not be avoidable with ECoG data and psychophysics tasks, but it makes interpretation of the proportions more difficult than the text suggests.

Minor(ish) points:

====================

lines 140--145: what about responses with correct location but incorrect emotion? Were they simply omitted from the analysis?

lines 165--180: T=200ms, but tau=? And why were 200ms windows chosen?

line 319: The reference to the toolbox borders on supplementary materials of the type that eNeuro (and JNeuro) really seems to hate

line 394: “non linear / non monotonic” -- with respect to what ordering? In terms of size of subset?

lines 424--432; line 447: the poorer clustering holds for the nearest neighbor algorithm, not necessarily in general. Even unsupervised classification methods have lots of assumptions! Addressing these assumptions / explaining why integrated information does better under these assumptions would be very interesting because it would get more at what integrated information is actually capturing (which is somewhat examined around lines 502--514.

line 495--496: “Although our investigation of this hypothesis cannot be posed as a test the integrated information theory per se” -- Yes.

line 497: “integrated information pattern in human visual cortex closely matched with psychophysically-established perceptual contents.” -- Where were the electrodes in the visual cortex? There are none in _primary_ visual cortex and the areas covered by several of the temporal lobe grids are getting close to *auditory* cortex. Arguably, you capture parts of the dorsal and ventral streams, but I would be more careful about language here (especially since the difference between the roles of these areas is what enables phenomena like blindsight).

lines 550--567: this would be a good point to discuss / contrast your approach to graph-theoretic/connectivity-based approaches (in ECoG, fMRI and to a lesser extent M/EEG).

lines 570--580: I'm not sure I follow the logic here, but it seems to have an implicit criticism to other decoding approaches, which made me think of Haxby's hyperalignment approach with its *abstract* neural code. Perhaps it would make sense to more explicitly contrast these approaches.

Figure 7 & 8: I'm not sure the graph presentation is the best way to present this data. Especially since the “The x and y coordinates here are assigned for visualization purposes.” I understand that the graphs provide a convenient way of showing relationships between partitions (which is very nice), but the it might make sense to have two panels, one with the graph representation and no integrated information depiction (or maybe depicted as color?), one with the various subsystems just ordered along the x-axis by degree and integrated information on the y-axis.

---------------

In this paper, authors aim at validating the integrated information theory (IIT), a theory of consciousness proposed by Tononi et al..

In order to achieve this goal, authors have used intracranial ECoG recordings in patients, during the presentation of different paradigm of visual stimulations. The authors show that the amount of integrated information patterns in neural activity (phi) is related to awareness. Furthermore, they also demonstrate that it is possible to cluster concepts based on the phi metric. Finally the authors showed that phi analysis gives better results than classical entropy and mutual information measures.

The article is not a full validation of IIT consciousness theory. The paradigm used in this study test one component of consciousness which is awareness (or conscious perception) of a visual stimulus. However the authors certainly provide some appealing evidence for IIT validation, with a hierarchical pattern of interactions between neural populations. The study is interesting, the methodology is solid, and the results are compelling.

Some points need to be clarified.

1- Are all the patients epileptic?

As epilepsy could affect neural network structure, and even interictal activity may modify the synchrony of distal neuronal groups, functional connectivity of distant neuronal groups, disturb information processing, and the overall excitability, two points need to be added.

i) please provide some clinical data in Table 1 (at least , which type of epilepsy).

ii) Would the method (phi analysis) also be valid in control, non epileptic-subject, recorded with non-invasive dense-EEG for instance? Or maybe the high number of channel provided by Ecog is required? Please discuss this point. To what extent the results could be affected, in theory, by all the potential structural and functional modifications mentioned in point 1? Please add it to the discussion.

2- Please provide neuroanatomical data in table 1 to indicate the location of electrodes (on fusiform gyrus, inferior temporal gyrus, angular gyrus, ...)

3- In the backward masking paradigm, what is the rational to use different emotions, instead of showing neutral images? Is it useful for your analysis? No data are presented regarding Phi calculated from distinct emotional stimuli.

4- Results showed in Fig 6,7,8 and 9 were obtained from the same patient (N{degree sign}153) with ECOG placed likely onto the right fusiform face area (FFA). And PHI analysis provides excellent results when faces were shown. What happens if the same analysis is done from signals recorded from electrodes located on brain areas which are not involved in face processing (red yellow spots on the superior temporal lobe, fig 5) ?

5- Does all the subject recorded onto fusiform gyrus ? If not, how does it affect the average results obtained from 6 patients (fig 10,11) ?

6- Please add a sentence regarding the patient's informed consent and its approval.

7- In terms of manuscript's structure:

Results section should be re-worked so that all of the Methods-related information provided there should be moved into the Methods section. Indeed, the authors present and describe many concepts in this section whereas it should not be presented here (see the subsections « Behavior », or « Measuring Integrated Information » in the Methods section for example). Therefore, the Results section as it is in the submitted paper is difficult to follow which somehow distracts from the main message. Actual results begin p13 l 350 (Fig5).

1- Minor points:

-ID numbers for patients are not necessary. Use simpler P1, P2, P3,...instead.

-Fig 1 is noticed left / right in the legend but 1A/B in the text

-In table 1 channels is the number of electrode contacts?

- p. 4, line 96, the sentence « Subjects, experimental paradigms... » should be reworked.

- p.18 line 495, there is a missing « of » between « test » and « the integrated ».

## Author Response

Authors: We greatly appreciated the reviewers' helpful comments and suggestions for our manuscript. Please find below our point-by-point replies to each comment. Our responses to the reviewers are indented with respect to the reviewers' comments [different formatting was used in the original communication].

Synthesis of Reviews:

Significance Statement Comments for Author (Required):

Editor: In this paper, authors aim at validating the integrated information theory (IIT), a theory of consciousness proposed by Tononi et al.The article is not a full validation of IIT consciousness theory. The paradigm used in this study test one component of consciousness which is awareness (or conscious perception) of a visual stimulus. However the authors certainly provide some appealing evidence for IIT validation, with a hierarchical pattern of interactions between neural populations. The study is interesting, the methodology is solid, and the results are compelling.

Authors: We thank the reviewers for positive assessment of our paper.

Comments on the Visual Abstract for Author (Required):

-

(Reviewer 1)

Synthesis Statement for Author (Required):

Reviewer: Using ECoG data, the authors examine which information-theoretic measure -- entropy and mutual information from classical information theory and integration information from a more contemporary source -- best tracks “conscious” information processing in the brain as indexed by detection of target stimuli in various masking paradigms. They argue that the partitioning scheme inherent in integration information, which is designed to capture the hierarchical structure in complex signals, parallels the hierarchical structure of conscious experience. Therefore, they argue, the better decoding performance (model fit) of integrated information on their conscious information processing task(s) shows that integrated information best tracks the emergence of conscious information processing.

I find the performance of integrated information compared to more classical measures (entropy and mutual information) to be very interesting, especially as integrated information is a function of entropy of the subsets / partitions of the data. However, I am not convinced that the present manuscript addresses the central issue of its current formulation, namely the “significant role of locally integrated information for understanding the neural substrate of conscious experience” (from the abstract) or the “phenomenon occurring in brains that is critical for consciousness” (from the significance statement).

Authors: We claimed the role of integrated information by the comparison between structures due to integrated information and non-integrated information measure (e.g., entropy and mutual information). Because we used “local” 4 neighboring bi-polar re-referenced voltages, we claimed the role of “locally integrated information”. From the rest of the comments, however, we surmise the the reviewer had an issue with the term “consciousness experience” or “consciousness”. As we describe below, our paper only speaks about “conscious perception of an object”. Accordingly, we modified these terms into “conscious percept”.

Reviewer: There seem to be two motivating ideas behind the claim that the authors are capturing (the emergence of) consciousness: (1) their dependent measure is “conscious awareness of the percept” based on behavioral performance in a detection task and (2) the parallel structure of integrated information to the structure of conscious perception. Let me address these in turn.

I'm not sure that detection performance is sufficient for consciousness. An amoeba can detect elements of its environment and produce an appropriate behavioral response without anything approaching a nervous system. Accurately assessing the facial expression as opposed to simple target detection does address some of this -- it would seem that facial expressions require something like consciousness but then again, non-human primates whose conscious nature is more difficult to assess are also capable of processing facial expressions. The difficulty here may very well lie in defining what consciousness is, which brings me to the other potential motivating idea, the parallel structure of integrated information to the structure of conscious perception.

Authors: Over last 30 years of consciousness research has converged on the agreement that any types of detection performance per se is NOT sufficient for consciousness. The prime example of non-conscious detection is blindsight, as the reviewer points out. Accordingly, we did not use detection as a criterion for conscious perception. Instead, our criteria for conscious perception differs for each experiment.

In the CFS task, we defined conscious perception of face as “correct detection which accompanies with high confidence”, implying accurate conscious access and high metacognition. This is one of the most solid criterion available in the literature [Seth et al 2008 TICS]. Among the three tasks, this is also most stringent. In the Backward Masking task, the criterion was less direct. However, we required “correct spatial localization (4AFC) and facial emotion recognition (3AFC)”. While above chance performance in non-conscious emotion recognition has been reported to in healthy (Jolij & Lamme 2005 PNAS) and blindsight subjects (Pegna et al 2005 Nat Neuro), we do not know a case of non-conscious perception of both location and emotion. Finally, our Unmasked tasks presented stimuli for a long time (500 ms) with no masks, no expectation manipulation, and no attention demanding task. In such a situation, there is no reason and evidence to expect that subjects did not see the unmasked images. Thus, we assumed subjects are conscious of the images in UNM task.

Our intention of this study is to take the CFS trials as the primary measure of ‘conscious face perception’ and use the BM and Unmasked trials, with less strict definition of ‘conscious face perception’, as secondary data, which allows us to test the generality of our claims. We indeed find that phi* structure in the three paradigms are similar when faces are consciously perceived (Figure 8&9, Video 2).

We have expanded the text in the Psychophysics Methods section to make our intentions clearer: the CFS task is our primary test, and the BM/UNM tasks are secondary.

Pages 5

“The primary task was continuous flash suppression (CFS), where a target face stimulus is presented to one eye while colorful Mondrian (shape noise) images are continuously flashed to the corresponding position in the other eye (Tsuchiya & Koch, 2005)”

Pages 6-7

“Of these three tasks, only the CFS included a metacognitive judgment. Within a subset of CFS trials, subjects received physically identical visual input, yet they consciously experienced seeing the face target only on some of the trials. By dissociating stimulus parameters from conscious perception, this type of paradigm has been successfully used to isolate the neural correlates of consciousness (Koch, 2004). We include the other tasks as complementary to the CFS task to test the generality of our claims across different tasks. With regard to our classification of BM trials: non-conscious emotion discrimination has been reported in blindsight (Pegna, Khateb, Lazeyras, & Seghier, 2005) and in healthy subjects (Jolij & Lamme, 2005). However, we required correct discrimination of both location and emotion of the face targets, reducing the likelihood of including trials with a perceptually invisible face. In the UNM task, we presented the stimuli for a relatively long duration (500ms) at fixation with no masks, no misdirection of expectation, and no attention demanding tasks. In such a situation, target perception is extremely unlikely to be non-conscious.”

Reviewer: The authors argue that conscious experience has an intrinsic, integrated, and hierarchical structure. However, this cannot be the defining quality of conscious experience because this type of structure is found everywhere. In neuroscience, this is found e.g. in Friston's predictive coding framework (which I would suggest that the author's examine for useful parallels and connections to integrated information), yet predictive coding is not a theory of consciousness -- one of the classic examples for predictive coding is the MMN, which can be elicited from coma patients.

Authors: A description of conscious experience as intrinsic, integrated and hierarchical is indeed rather general, so that something might satisfy these properties without being conscious. IIT explains whether an entity is conscious or not according to the another property, called “exclusion” [Oizumi et al 2014 PLoS Comp]. However, predictions related to “exclusion” is most difficult to assess in our paradigm. Thus, while we made an effort to discuss this issue (see below), we would like to keep the discussions on this issue short (as the Discussion is already getting very long).

As to Friston's framework, thank you for suggestion for the formal comparison with the predictive coding framework. In fact, the last author is working on this issue with one of the proponents of predictive coding theory, Jakob Hohwy, who is in the same institution (Monash University). While a serious comparison between IIT and predictive coding is beyond the scope of the current manuscript, we made made an effort in pointing out a critical comparison in Discussion.

Although there are apparent parallels between predictive coding and IIT (e.g., the emphases on feedback and hierarchical structure), they are actually quite different. For example, the treatment of “intrinsicality” is very different. IIT tries to derive all relevant quantity from purely system's own intrinsic viewpoint. However, predictive coding often relies on extrinsic viewpoint. In particular, when predictive coding talks about “feedback” and “feedforward”, it is very unclear how the system (or neuron) itself can possibly know what is feedback and what is feedforward. The ‘hierarchy’ in a predictive coding model is in the eye of the extrinsic observer - it does not seem to be an intrinsic property to the system. (Similarly, from intrinsic viewpoint, it is totally unclear what it means to “predict” or “produce prediction error” for recurrent connections).

Also, while we do not explore the notion of boundary in our study, the absence of an “exclusion”-like concepts in predictive coding starkly distinguishes it from IIT. In IIT, there is the notion of a “complex” defining the boundary between conscious and non-conscious processing - any system extending beyond the complex is excluded from consciousness. In IIT, the non-conscious MMN would correspond to processes outside the boundary of the complex. In predictive coding, the nearest corresponding concept would be the “Markov blanket” (Friston 2013 J R Soc Int). However, the straightforward reading of Markov blanket is the boundary between environment and the agent. If it is the case, the non-conscious MMN, for example, should be a process inside of the Markov blanket. As far as we can tell, there is no explanation in predictive coding that distinguishes non-conscious from conscious processing.

To keep the paper short as the reviewer/editor recommend, we included a shorter version of the above paragraphs as follows:

Page 19

“The idea of an informational hierarchy having a close relation to the substrate of consciousness may seem familiar - computational theories (e.g. Marr, 1982) and more recently predictive coding theories (e.g. Hohwy, 2013) of the mind heavily rely on the notion of hierarchical substrates. In these schemes, a ‘high-level’ neuron may receive inputs from ‘low-level’ neurons, and feed back onto the lower level to adjust the sensitivity of its inputs. However, the neurons themselves cannot know their location within the hierarchy. Likewise, they cannot know which inputs are from high- or low-level neurons. The hierarchy is only there in the description of the system by the experimenters. IIT does not take on this kind of extrinsic viewpoints: the hierarchical structure of a system emerges intrinsically as the integration of the system's parts. Thus we did not assume any hierarchy of ECoG channels, but instead extracted hierarchical patterns intrinsic to the activity across those channels. This is an important property afforded by IIT that does not seem to be obtained from hierarchical models of information processing in the brain.”

Reviewer: (And to fully argue integrated information being isomorphic to conscious experience, the authors would need to demonstrate that its decoding performance is bad / worse than classical measures for unconscious states / behaviors. Maybe something demonstrating that integrated information processing tracks P300 related activity but not MMN?)

Authors: This is an excellent suggestion! Unfortunately, we do not have the data to test such situation (clear neural activity that represent non-conscious processing). While our results on the CFS and BM trials where subjects reported not to see faces are consistent with this idea, the electrodes that we examined were located in the middle to the high level visual areas, thus we did not see clear neural activity that is clearly dissociated from conscious perception. We have expanded on this point at the end of Discussion as follows:

Page 21:

“We see several immediate avenues of development that should follow from our study; first, we would propose that new tests of the Φ-pattern concept should include conditions of constant stimulation under varying levels of consciousness (e.g. comparison of Φ-patterns during wakefulness and anesthesia) - similar types of Φ-pattern should be observed during dream states as during wakefulness, but different types (with much lower Φ magnitude) should be observed during dreamless sleep or deep anesthesia. “

Reviewer: Again, I find the integrated information and its performance quite interesting, but I'm not sure the present paper demonstrates that it indexes conscious experience -- perhaps “perception” and maybe “conscious perception” but not “conscious experience”. My concrete suggestion would be to focus on an experimental validation of the integration information as capturing some aspect of perception and neural processing -- notice the lack of “conscious”. In the discussion, it would be appropriate to tie into other literature and note that the present data provide are compatible with (and perhaps provide converging evidence for) a theory of consciousness based on integrated information.

Authors: While we agree that our study did not rule out that integrated information does NOT correlate with non-conscious perception as you suggested in the previous point, we did manipulate the conscious perception of faces when identical physical input is provided to the visual system in the CFS task (Figure 11C being the purest example of this). Such tasks, keeping the physical stimulus the same and varying the conscious perception, have been traditionally regarded as the best practice for isolating the neural correlates of consciousness (e.g. Crick & Koch 2003 Nat Neuro). We admit that we were ambiguous as to what we mean by “conscious experience” and how it differs from “conscious perception of an object”. In the revision, we made the distinction clearer by referencing the former as “conscious experience as a whole” and we made it clear that our paper only addresses “conscious perception”. We believe this distinction should now be clear throughout the paper.

Reviewer: (I'm also curious as a thought experiment what would happen with this measure in blindsight patients, who can avoid obstacles despite having no / a very different conscious visual perception of them.)

Authors: We agree this would be an important test - if high-level visual cortex still generates Φ* patterns that map on to stimuli to which the observer claims to be blind, this would be very bad news for our hypothesis. Note, however, that a full validation of IIT would include the identification of “complex”, a subset of neurons that attains local maximum of integrated information; observed ‘local’ patterns of Φ that correlate with conscious perception would need to belong to this complex. At the moment, identification of the complex is extremely computationally demanding and we simply cannot do it with current available tools. If we could identify the complex, we predict that non-conscious object recognition related activity in blindsight, integrated or not, would be happening outside of the complex. We mention this in Discussion as follows:

Page 21

“IIT would require that for a local Φ pattern to be a part of conscious experience as a whole, it must be a part of a much larger pattern extending across multiple cortical regions. Our procedure did not measure this superstructure, called a complex in IIT (Tononi et al., 2016), another reason that our procedure cannot be taken as a direct test of IIT.”

Reviewer: I found the first half of the manuscript to be fairly well written. There is a fair amount of mathematics, which will be challenging to some readers, but the authors do a decent job in their exposition. The organization isn't always ideal, but this is not particularly problematic in the first part of the manuscript.

The writing really breaks down around the start of the Results section, where large portions seem to be nearly exactly duplicate previous paragraphs. The clarity of the mathematical exposition also drops at this point. The authors should re-work this section so that there is less repetition and more clarity and “flow”. This could also potentially shorten the manuscript a fair amount -- short is not always better, but short will generally increase your readership. As I think this needs a fair amount of revision, I won't list individual edits.

Authors: Thank you pointing this out. We revised the Results section by removing repetition and improved the readability by shortening it.

Statistical comment (in response to the eNeuro prompt “Is the statistical analysis correct?”):

==========================================================

Reviewer: The statistical analysis using an ANOVA over z-transformed ROC AUC may be technically correct (I'm not entirely sure). I'm assuming that the authors performed a Fisher z-transformation to avoid issues with boundedness inherent in the ROC AUC measure, but “z-transformed” (i.e. without “Fisher”) can also refer to the standardization/scaling procedure where data are expressed as standard deviations away from the mean. The latter transformation would not be appropriate. Moreover, the Fisher z-transform does not address the boundedness issue for ROC AUC because it is still bounded at zero. For correlations, which go from -1 to 1, this is not the case; but AUC only goes from 0 to 1. Perhaps a simpler method than ROC AUC + Fisher transform would be to use d-prime. I find explicit regression techniques to almost always be better than ANOVA procedures.

Authors: Thanks for catching this. We realize our previous description on ‘z-transformation’ was ambiguous. The inverse cumulative normal distribution is usually called the Z function. To avoid confusion with either Fisher's z or z-transformation (subtraction of mean and division by variance), we now call it as zAUC value. zAUC=0 when AUC=0.50 and zAUC=1.0 when AUC=0.84, etc. This transform is appropriate for AUC which varies from 0 to 1. Now we clarify it in the last sentence of the methods as:

Page 12:

“For statistical analysis, each AUC value was converted into a zAUC score through the inverse cumulative normal distribution, which removes the 0-1 bounds of the AUC value and is more appropriate to ANOVA.”

Reviewer: I'm also a bit concerned by the uneven distribution of trial numbers (e.g. in Fig 4) when calculating proportions. I realize this may not be avoidable with ECoG data and psychophysics tasks, but it makes interpretation of the proportions more difficult than the text suggests.

Authors: While we agree with a potential concern on the uneven distribution of trial numbers, our usage of the cross-validation procedure should reveal any bias. We now explicitly point it out in the Methods as follows:

Page 11

“Cross validation removed any possible bias due to unequal number of trials due to percept.”

As we expected, we did not find any deviation from the chance level at the pre-stimulus time period (Figure 11a), confirming the absence of any bias introduced by the uneven trial numbers.

Minor(ish) points:

====================

Reviewer: lines 140--145: what about responses with correct location but incorrect emotion? Were they simply omitted from the analysis?

Authors: That is correct. We now explicitly state it.

Page 6

“Trials where the subject correctly identified the location but not the emotion were excluded from analysis for this study.”

We omitted these trials since we cannot plausibly suppose that subjects consciously saw these faces in the same way as those for which both judgments were correct.

Reviewer: lines 165--180: T=200ms, but tau=? And why were 200ms windows chosen?

Authors: Like the searchlight location, we also found the value of Tau for each subject by looking at what Tau value maximized the evoked Φ*. We chose 200 ms considering the trade off of temporal resolution and relevant time-scale of conscious perception. We now explicitly state it

Page 7

“T=200ms throughout the paper; we found 200ms to be a realistic range of the temporal window for conscious perception and a good compromise between better temporal specificity and better estimation of the covariances.”

Reviewer: line 319: The reference to the toolbox borders on supplementary materials of the type that eNeuro (and JNeuro) really seems to hate

Authors: We removed the reference to the toolbox.

Reviewer: line 394: “non linear / non monotonic” -- with respect to what ordering? In terms of size of subset?

Authors: Yes-This has been clarified in the text.

Page 13

“The 4-channel Φ* pattern in Figure 7 illustrates an important property of Φ* that distinguishes it from H and I: nonlinearity and nonmonotonicity with respect to the size of the subsystem. Here the 3-channel subsystem ACD attains the highest Φ* of all 11 possible subsystems. The graph illustrates how subtracting from or adding to a subsystem can reduce the integrated information. The Φ* of ABCD is less than that of ACD because there is a relatively weak interaction between B and ACD. In this case, the minimum information partition (MIP) for ABCD is between B and ACD.”

Reviewer: lines 424--432; line 447: the poorer clustering holds for the nearest neighbor algorithm, not necessarily in general. Even unsupervised classification methods have lots of assumptions! Addressing these assumptions / explaining why integrated information does better under these assumptions would be very interesting because it would get more at what integrated information is actually capturing (which is somewhat examined around lines 502--514.

Authors: It is certainly true that different classification methods may give different results. We tried to keep things simple by using as simple a method as possible. We added the following in Discussion about what it is that Φ* is probably doing that improves its mapping to conscious perception.

Page 17

“Another way to understand the advantage of Φ* is as noise reduction: each step in the derivation of Φ* involves not the construction of new information from the neural data, but ‘deletion’ of noise, which improves the performance of a simple clustering algorithm. When mutual information is computed from entropy, it is by subtracting the part of the system noise that is not carried across the time lag τ; when integrated information is computed from mutual information, it is by subtracting the part of the system noise that is only within parts of the system. Derivation of Φ* can then be seen as reduction of the system noise to only those portions that persist across the time lag, and across the parts of the system. The remaining noise that persists across system parts and across time corresponds to variation in conscious percepts.”

Reviewer: line 495--496: “Although our investigation of this hypothesis cannot be posed as a test the integrated information theory per se”

line 497: “integrated information pattern in human visual cortex closely matched with psychophysically-established perceptual contents.” -- Where were the electrodes in the visual cortex? There are none in _primary_ visual cortex and the areas covered by several of the temporal lobe grids are getting close to *auditory* cortex. Arguably, you capture parts of the dorsal and ventral streams, but I would be more careful about language here (especially since the difference between the roles of these areas is what enables phenomena like blindsight).

Authors: We modified the language as follows:

Page 17

“Although our investigation of this hypothesis cannot be posed as a test of the integrated information theory per se (See below), we did find that the integrated information pattern in human cortex closely matched with psychophysically-established perceptual contents. “

We also added information in Table 1 describing the anatomical location of each “max evoked Φ*” electrode set (those included in the main analyses). While most are unambiguously in visual cortex, the supramarginal gyrus shows up in multiple subjects (we prefer however not to speculate on the detailed physiological underpinnings of the observations, since we do not have sufficient data to talk about different e.g. perceptual/cognitive functions of the recorded ECoG channels).

Reviewer: lines 550--567: this would be a good point to discuss / contrast your approach to graph-theoretic/connectivity-based approaches (in ECoG, fMRI and to a lesser extent M/EEG).

Authors: We agree that it would be good to discuss this issue, but we also feel that our discussion is getting longer in answering other points. We added the following sentences in the discussion to point to similar-in-spirit current approaches, although we prefer to keep things short on these points.

Page 20

“At the same time, current perspectives on neural processing do seem to be moving towards high-dimensional descriptions of how connected brain systems accomplish the jobs of consciousness, perception, and cognition. Looking at synapse-level connectivity of large groups of single neurons, (Reimann et al., 2017) have recently shown how neural systems can be understood in terms of their topological features, treating them as sets of overlapping groups of connected neurons. (Lord, Stevner, Deco, & Kringelbach, 2017) have described how a different set of methods to distinguish ‘integration’ (the holistic property of a neural system) from ‘segregation’ (the way the parts are differentiated from one another) can be used to understand psychiatric disorder and other aspects of whole-brain function. So, even detached from IIT, the notion of an intrinsic hierarchy of integrated neural structures may prove to be extremely fruitful for future neuroscience research.”

Reviewer: lines 570--580: I'm not sure I follow the logic here, but it seems to have an implicit criticism to other decoding approaches, which made me think of Haxby's hyperalignment approach with its *abstract* neural code. Perhaps it would make sense to more explicitly contrast these approaches.

Authors: We modified the paragraph to make our argument clearer:

Page 20

“As we emphasized in Results, we intentionally avoided “optimizing the classifier” by finding the best weights to achieve higher AUC. While such decoding analyses have their own advantages in the search for neural correlates of consciousness (NCCs) (for our own effort on the same dataset see [redacted for eNeuro double-blind review]), there is an important caveat in such an approach: the assumption of the decoder. Even if one can perfectly identify conscious perception based on patterns of neuronal activity, the interpretation of a vector of regression weights (for example) is entirely dependent on the presence of a decoder - usually a tool in the hands of the experimenter. What we learn from this kind of analysis is that the information necessary for consciousness is available in a certain brain area. But there is no clue as to what it should feel like - why should one vector of weights feel so different from another? A hierarchical structure of integrated information does not pose the same problem, because it is hypothesized a priori as the structure of experience, serving as a theoretical basis for the experiment.”

As this is not really directed to a particular decoding approach, including Haxby's, we prefer not to refer to a specific study here. In fact, we ourselves also use decoding approach in other papers for different purpose, as we admit in the paper.

Reviewer: Figure 7 & 8: I'm not sure the graph presentation is the best way to present this data. Especially since the “The x and y coordinates here are assigned for visualization purposes.” I understand that the graphs provide a convenient way of showing relationships between partitions (which is very nice), but the it might make sense to have two panels, one with the graph representation and no integrated information depiction (or maybe depicted as color?), one with the various subsystems just ordered along the x-axis by degree and integrated information on the y-axis.

Authors: We are presenting the results in this graph form to emphasize the compositional structure of the information patterns. We've rewritten the text as these data are introduced as follows:

Page 13

“The Φ* pattern is not just a vector of integrated information values; each value has a particular relation to some of the others, in the manner of an inclusion or intersection relation. This property of the Φ* pattern distinguishes it from more orthodox measures that aim to reduce neural activity to independent components. The inclusion relations in a Φ* pattern can be illustrated in the manner of a Hasse graph (Figure 7A)”

Of course when the classification is done, the patterns are treated simply as vectors of numbers, and some of their meaning is lost. Ideally in the future, we will know more about what each neural channel is doing (what it is selective for), and then the compositional structure of a Φ pattern can be compared part-for-part with the compositional structure of the corresponding percept. For now, we just want to give the implication of the potential for this method. This idea has been expanded as follows:

Page 19

“For example, suppose that connected but distinct neural populations encode the presence of particular facial features (an enormous simplification; see e.g. (Chang & Tsao, 2017; Freiwald et al., 2009; Henriksson, Mur, & Kriegeskorte, 2015)), with one population encoding eyes (population E), another encoding nose (population N), another encoding mouth (population M), and another encoding a group of features (population G). When a face is seen, each population will be active, but the features must be somehow bound together in a whole in order to correspond to the holistic experience of a face composed of features; that is, there must be a physical substrate that reflects the integration of facial features. For such a system of neural populations, when a face is seen, activity in mouth might be moderately integrated with activity in nose (i.e. Φ*{M,N}<0), nose with eyes (Φ*{N,E}<0), all three features together (Φ*{M,N,E}<0), and any of these should be strongly integrated with the group population (Φ*{E,G}<0, Φ*{M,G}<0, etc); meanwhile, mouth and eyes populations may not be integrated at all (i.e. Φ*{M,E}≈0). The Φ* pattern generated by these populations during visual experience of a face would therefore have a particular structure isomorphic to the complex experience. While we have no way of ascribing particular functional roles to the ECoG channels in our dataset, this scenario suggests a plausible interpretation for Φ* patterns.”

For comparison, here is a set of bar plots of the Φ* patterns of figure 8. While it provides easier representation of the magnitude of Φ* for each mechanism, it is difficult to find the similarity of the meaningful patterns. Thus, if the editor and reviewers are OK with it, we would like to keep the original format.

[The figure provided here to the reviewers can be found at https://figshare.com/s/36477e78692cc219435c]

---------------

(Reviewer 2)

Reviewer: In this paper, authors aim at validating the integrated information theory (IIT), a theory of consciousness proposed by Tononi et al..

In order to achieve this goal, authors have used intracranial ECoG recordings in patients, during the presentation of different paradigm of visual stimulations. The authors show that the amount of integrated information patterns in neural activity (phi) is related to awareness. Furthermore, they also demonstrate that it is possible to cluster concepts based on the phi metric. Finally the authors showed that phi analysis gives better results than classical entropy and mutual information measures.

The article is not a full validation of IIT consciousness theory. The paradigm used in this study test one component of consciousness which is awareness (or conscious perception) of a visual stimulus. However the authors certainly provide some appealing evidence for IIT validation, with a hierarchical pattern of interactions between neural populations. The study is interesting, the methodology is solid, and the results are compelling.

Some points need to be clarified.

1- Are all the patients epileptic?

As epilepsy could affect neural network structure, and even interictal activity may modify the synchrony of distal neuronal groups, functional connectivity of distant neuronal groups, disturb information processing, and the overall excitability, two points need to be added.

i) please provide some clinical data in Table 1 (at least , which type of epilepsy).

Authors: We have added information about the epilepsy focus in each subject (Table 1). In each subject, the seizure focus was not in the same location as the ‘max evoked Φ’ locations we used for our primary analyses. We did not see much signs of interictal activity during our experiments. Thus we have not attempted to include this information in our analyses.

Reviewer: ii) Would the method (phi analysis) also be valid in control, non epileptic-subject, recorded with non-invasive dense-EEG for instance? Or maybe the high number of channel provided by Ecog is required? Please discuss this point. To what extent the results could be affected, in theory, by all the potential structural and functional modifications mentioned in point 1? Please add it to the discussion.

Authors: Our group is currently applying the same analytical procedure to various data sets to address the exactly this issue. However, the results are not conclusive at this point and we prefer not to make any solid statements here. Instead we wrote in Discussion

Page 18

“A more theoretical caveat is that while we are clearly limited in measuring integrated information across neural populations as measured by ECoG electrodes, this does not mean that the ‘true’ underlying integrated information structure involves populations as basic units; the integrated information structure corresponding to conscious experience may exist at a much finer scale such as just a few neurons or other more local physical structure (Barrett, 2014; Hoel, Albantakis, Marshall, & Tononi, 2016; Tononi, Boly, Massimini, & Koch, 2016). We must therefore be cautious in interpreting the meaning of Φ* patterns obtained from such coarse recordings as ECoG electrodes which sum responses over many tens of thousands of neurons; accordingly, our procedure is not a direct test of the IIT. “

Reviewer: 2- Please provide neuroanatomical data in table 1 to indicate the location of electrodes (on fusiform gyrus, inferior temporal gyrus, angular gyrus, ...)

Authors: We have added in Table 1 the locations of the max evoked Φ* regions that we used for the non-searchlight analyses.

Reviewer: 3- In the backward masking paradigm, what is the rational to use different emotions, instead of showing neutral images? Is it useful for your analysis? No data are presented regarding Phi calculated from distinct emotional stimuli.

Authors: The BM experiment was devised for a study of interaction of emotion processing and consciousness, not for a study of identifying neural correlates of consciousness. We recruited these data because of the similarity of the stimuli (face targets) to the main CFS task. With some added text in Methods, we made it clear that these were not the ideal set of data, and that face emotion per se was not considered as a variable for our study.

Page 6

“We include the other tasks as complementary to the CFS task to test the generality of our claims across different tasks.”

Page 6

“Trials where the subject correctly identified the location but not the emotion were excluded from analysis for this study. We did not include the face emotion as an independent variable in any analyses for this study.”

Page 7

“we required correct discrimination of both location and emotion of the face targets, reducing the likelihood of including trials with an perceptually invisible face”

Reviewer: 4- Results showed in Fig 6,7,8 and 9 were obtained from the same patient (N{degree sign}153) with ECOG placed likely onto the right fusiform face area (FFA). And PHI analysis provides excellent results when faces were shown. What happens if the same analysis is done from signals recorded from electrodes located on brain areas which are not involved in face processing (red yellow spots on the superior temporal lobe, fig 5) ?

Authors: As requested, we show the results for the maximum evoked Φ region (the red diamond in Figure 5) for S153's lateral electrode grid, in the format of Figure 8. These channels are over S153's supramarginal gyrus. Because this set evoked the maximum phi in our criterion, it is the same system used for subject 153's temporal condition in Figures 11-13 (noted Fig.11c ‘153(L)’) and for the statistical analysis. It seems this system does not reflect the percept categories at all, though it generates high value of phi. Note that from Fig 11c, I or H structure obtained from this set also do not classify conscious percepts. It is plausible that this set is not a part of “complex” as we explained above. This of course remains a conjecture at this point, because we do not at present have a computational procedure to estimate the integrated complex over the whole channel set (if we tried to apply the technique in the paper to all the data channels, it would probably take millions of years to complete the computation).

[The figure provided here to reviewers can be found at https://figshare.com/s/1f3af19a33d685fc15a6]

As Figure 10 shows, when classiifcation is good, it is with the phi pattern, but not with I or H. But usually, no pattern is a good classifier since most cortical areas are not encoding the stimulus conditions. We believe it is better to keep this paper not longer than the current format as the reviewer and editor recommends. We are happy to provide these examples in the paper where phi analysis does not work if reviewers believe these are essential

Reviewer: 5- Does all the subject recorded onto fusiform gyrus ? If not, how does it affect the average results obtained from 6 patients (fig 10,11) ?

Authors: Each subject's electrode setting was unique but 5/6 that we studied did have ventral electrodes. Table 1 now shows the location of each subjects ‘evoked Φ maximum’ - in 4/5 of the subjects with ventral electrodes, the maximum was indeed over the fusiform gyrus.

Reviewer: 6- Please add a sentence regarding the patient's informed consent and its approval.

Authors: Thank you for catching this. This has been fixed.

Reviewer: 7- In terms of manuscript's structure:

Results section should be re-worked so that all of the Methods-related information provided there should be moved into the Methods section. Indeed, the authors present and describe many concepts in this section whereas it should not be presented here (see the subsections « Behavior », or « Measuring Integrated Information » in the Methods section for example). Therefore, the Results section as it is in the submitted paper is difficult to follow which somehow distracts from the main message. Actual results begin p13 l 350 (Fig5).

Authors: Thank you for pointing this out. We have fixed a lot of these problems, apologies for the confusion.

Reviewer: 1- Minor points:

-ID numbers for patients are not necessary. Use simpler P1, P2, P3,...instead.

Authors: Since each subject participated in many experiments, which are described in many papers from overlapping groups of researchers [eg Tsuchiya 2008 PLoS ONE, Kawasaki et al 2011 JCogNeuro, Baroni et al 2016 BioRxiv], we would like to keep consistent ID numbers across all publications.

Reviewer: -Fig 1 is noticed left / right in the legend but 1A/B in the text

Authors: Fixed

Reviewer: -In table 1 channels is the number of electrode contacts?

Authors: Yes, we changed the label for clarity

Reviewer: - p. 4, line 96, the sentence « Subjects, experimental paradigms... » should be reworked.

Authors: Fixed

Reviewer: - p.18 line 495, there is a missing « of » between « test » and « the integrated ».

Authors: Fixed
